# YTHDC1 Orchestrates Glucose and Glutamate Rewiring to Overcome Lethal Metabolic Stress in Triple‐Negative Breast Cancer

**DOI:** 10.1002/advs.77492

**Published:** 2026-08-30

**Authors:** Zheng‐Hao Lai, Yi Wang, Na‐Na Li, Ling Zeng, Renzhong Liu, Xue Li, Cheukfai Li, Xin‐Yu Ma, Wenkui Fu, Yingsen Tang, Jinjin Chen, Ning Liao, Man‐Li Luo

**Affiliations:** ^1^ Medical Research Center, Sun Yat‐Sen Memorial Hospital Sun Yat‐Sen University Guangzhou China; ^2^ Guangdong Provincial Key Laboratory of Malignant Tumor Epigenetics and Gene Regulation, Sun Yat‐Sen Memorial Hospital Sun Yat‐Sen University Guangzhou China; ^3^ Wuxi Hospital Affiliated to Nanjing University of Chinese Medicine Wuxi China; ^4^ Department of Breast Surgery Guangdong Provincial People's Hospital and Guangdong Academy of Medical Sciences Guangzhou China

**Keywords:** disulfidptosis, m6A, metabolism, triple‐negative breast cancer, YTHDC1

## Abstract

Developing effective therapies for triple‐negative breast cancer (TNBC) requires the identification of key molecular regulators. Here, among the major m6A modulators, we find that only the reader YTHDC1 is specifically overexpressed in TNBC and correlates to unfavorable prognosis. Mechanistically, YTHDC1 orchestrates glucose and glutamine metabolism in an m6A‐dependent manner to confer robust adaptability to lethal metabolic stress in TNBC cells. It not only stabilizes GLUT3 mRNA to sustain glucose uptake and NADPH production, but also reduces the mRNA stability of ATF4, thereby suppressing the overexpression of the cystine/glutamate antiporter SLC7A11 to prevent excessive cystine uptake and glutamate export. YTHDC1 knockdown simultaneously disrupts glucose metabolism and upregulates SLC7A11, triggering disulfidptosis in TNBC cells in vitro and in vivo. Leveraging this YTHDC1 knockdown‐induced metabolic vulnerability, we develop an ATF4 mRNA‐targeted nanotherapy, which delivers mRNA directly to the cytoplasm and bypasses m6A‐mediated destabilization by YTHDC1 in the nucleus. By promoting SLC7A11 expression, therapeutic overexpression of ATF4 sensitizes TNBC cells to disulfidptosis upon GLUT inhibitor BAY‐876 treatment, and to glutamate deprivation‐induced cell death upon GLS inhibitor CB‐839 treatment. Collectively, this work reveals YTHDC1 as a pivotal coordinator of the glucose‐glutamine‐cystine metabolic network and provides ATF4 mRNA nanomedicine‐based combination strategies to improve TNBC therapeutic efficacy.

## Graphical abstract

Lai et al. find that m6A reader YTHDC1 is specifically overexpressed in triple‐negative breast cancer (TNBC) and orchestrates glucose and glutamine metabolism in an m6A‐dependent manner to confer robust adaptability to lethal metabolic stress. Leveraging YTHDC1 knockdown‐induced metabolic vulnerability, an ATF4 mRNA‐targeted nanotherapy is developed to combine with GLUT inhibitor or GLS inhibitor to improve TNBC therapeutic efficacy.

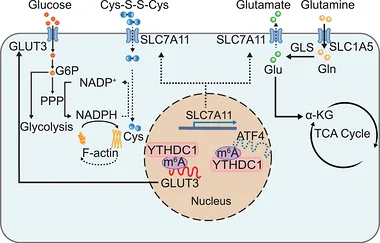

## 1 Introduction

Breast cancer has become the most prevalent malignancy in women worldwide [1]. Approximately 30% of triple‐negative breast cancer (TNBC) patients experience recurrence or metastasis, which is the leading cause of death in patients with TNBC [2]. While novel targeted therapies and immunotherapies have emerged for TNBC, improved outcomes are only observed in a subset of patients. Therefore, it is critical to identify the molecular vulnerabilities in TNBC and leverage them for the development of effective therapeutic strategies.

Cancer cells exhibit remarkable metabolic plasticity, supported by extensive crosstalk among metabolic pathways. This network flexibility enables them to rapidly rewire flux and engage alternative pathways upon metabolic stresses. Thus, therapeutic blockade of a single metabolic pathway in cancer cells exhibits limited efficacy, requiring optimized combination approaches that target multiple nodes to trigger metabolic catastrophe. Glucose and glutamine metabolisms are intricately connected and functionally cooperate to support biosynthesis and maintain redox balance. Glutamine is converted by mitochondrial glutaminases (GLS) to glutamate, which then can be processed to α‐ketoglutarate to enter the TCA cycle. Due to the Warburg effect, which diverts glucose away from oxidative phosphorylation (OXPHOS), many cancer cells are heavily reliant on glutamate to fuel the TCA cycle, making glutamine metabolism a key therapeutic target [3].

Intracellular glutamate is exchanged with extracellular cystine through an antiporter mechanism mediated by the amino acid transporter SLC7A11 (also known as xCT). Upon import, cystine (disulfide form) is rapidly reduced to the more soluble cysteine (thiol form). This reduction is NADPH‐dependent, directly coupling cysteine generation to the reducing capacity sustained by glucose flux through the pentose phosphate pathway (PPP). Excess cystine accumulation in the cytoplasm will create intense disulfide stress. Therefore, high SLC7A11 expression, which leads to elevated cystine uptake, together with glucose starvation that disrupts NADPH production, can trigger disulfidptosis, a novel form of cell death, characterized by aberrant disulfide bond formation in actin cytoskeleton proteins, NADPH depletion, and loss of cellular integrity [4]. Together, cancer cells encounter multiple forms of lethal metabolic stress that can induce cell death, including severe nutrient insufficiency (e.g., glucose and glutamine deprivation), energy crisis, and disulfide stress, etc. It is unclear whether there exists a master regulator that coordinates the metabolism processes to overcome these lethal stresses, and whether such a node presents an exploitable specific therapeutic target in TNBC.

Methylation accounts for more than 60% of RNA modifications, with N6‐methyladenine (m6A) as the predominant RNA methylation mark in eukaryotes [5]. The YTH domain ‐containing proteins, the primary m6A readers, critically regulate RNA metabolism, including RNA nuclear export, stability, and splicing [6, 7, 8]. Notably, through post‐transcriptionally dictating the expression of key metabolic genes (including MYC, GPT2, SLC1A5, and HK2), these m6A readers orchestrate metabolic reprogramming, thereby driving pivotal processes such as the glutaminolysis and Warburg effect in cancer [9, 10], highlighting the importance of m6A modification in tumor metabolic reprogramming and progression.

Here we report that among the major m6A modulators, only the reader YTHDC1 specifically overexpresses in TNBC and correlates with unfavorable prognosis. We reveal that YTHDC1 orchestrates glucose and glutamine metabolism to confer resistance to disulfidptosis in TNBC. On one hand, YTHDC1 stabilizes GLUT3 mRNA to sustain glucose uptake and NADPH production. On the other hand, YTHDC1 reduces the mRNA stability of ATF4, the major transcriptional factor of SLC7A11, thus preventing excessive expression of SLC7A11, resulting in lower cystine uptake and reduced glutamate export. YTHDC1 knockdown suppresses glucose uptake and upregulates SLC7A11 simultaneously, which triggers disulfidptosis of TNBC cells in vitro and in vivo. Furthermore, leveraging YTHDC1 knockdown‐induced metabolic vulnerability in TNBC cells, we develop an mRNA‐based nanomedicine of YTHDC1 downstream gene ATF4. Therapeutic overexpression of ATF4 promotes SLC7A11 expression, which predisposes TNBC cells to disulfidptosis under GLUT inhibitor treatment, and to severe intracellular glutamate deprivation‐induced cell death under GLS blockade. Together, our findings identify YTHDC1 as a key metabolic coordinator in TNBC and establish ATF4 mRNA nanotherapy as a promising approach to induce lethal metabolic reprogramming.

## 2 Results

### 2.1 m6A Reader YTHDC1 Rewires Glucose and Glutamine Metabolism in TNBC

**FIGURE 1:**
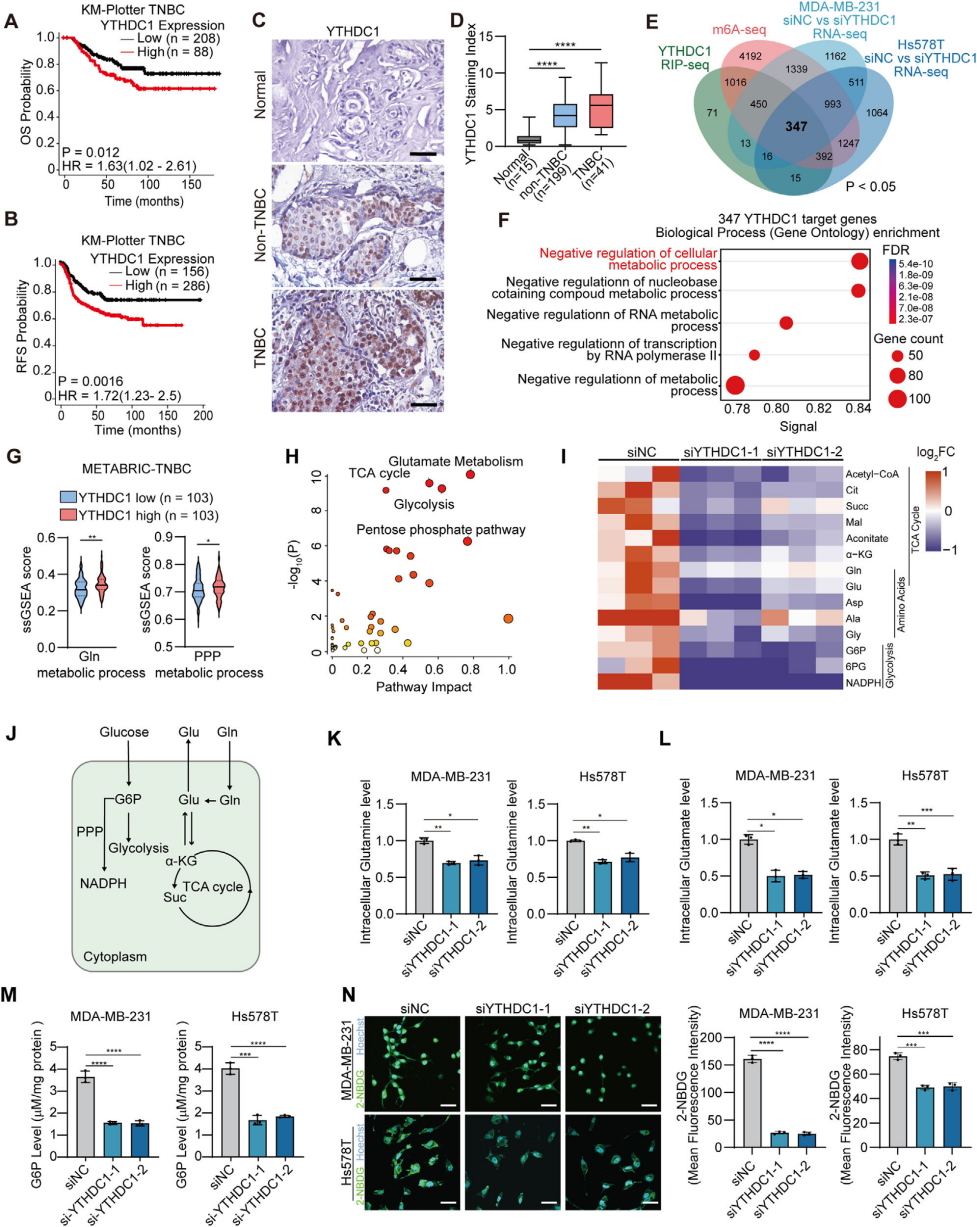
YTHDC1 rewires glucose and glutamine metabolism in TNBC. (A,B) Survival analysis of TNBC patients from Kaplan–Meier plotter database reveals that high expression of YTHDC1 is significantly associated with poorer overall survival (OS) (A) and progression‐free survival (PFS) (B). (C,D) Immunohistochemistry of YTHDC1 in tissue microarray (TMA) reveals that expression of YTHDC1 in TNBC is significantly higher than in non‐TNBC and normal tissue. Scale bars, 20 µm. (E) Venn diagram analysis integrating RNA‐seq (MDA‐MB‐231and Hs578T), m6A‐seq (MDA‐MB‐231), and YTHDC1 RIP‐seq (MDA‐MB‐231) data identifies 347 protein‐coding genes as high‐confidence m6A‐dependent targets of YTHDC1. Hypergeometric testing confirms that the three‐way intersection is highly significant (*p* < 0.05). (F) Gene Ontology (GO) analysis of the 347 candidate genes from (D) reveals significant enrichment in cellular metabolic process. (G) TNBC samples with high YTHDC1 expression exhibit higher ssGSEA scores of glutamine (Gln) metabolism and pentose phosphate pathways (PPP) signature compared to those with low YTHDC1 expression in METABRIC‐TNBC cohort (triple‐negative breast cancer cohort from the METABRIC database). (H) Metabolomic profiling in control and YTHDC1 KD MDA‐MB‐231 cells. Enrichment analysis illustrates that the citrate cycle (TCA cycle), glycolysis, and pentose phosphate pathways (PPP) are among the most significantly altered metabolic processes. (I) Heatmap depicting the relative abundance of metabolites from the pathways highlighted in (G) in YTHDC1 KD MDA‐MB‐231 cells. (J) A schematic diagram of the glucose and glutamine metabolism. The metabolites of these two processes were affected by YTHDC1 KD, as detected by metabolomic analysis. (K–M) Intracellular metabolite measurements show that YTHDC1 KD leads to decreased levels of glutamine (Gln) (K), glutamate (Glu) (L), and glucose‐6‐phosphate (G6P) (M). (N) YTHDC1 KD reduces glucose uptake of TNBC cells, as measured by the 2‐NBDG assay. Scale bars, 50 µm. Data are presented as mean ± SD of experimental triplicates (D,J–O). *p* values were assessed with Kaplan–Meier survival analysis (A,B), and two‐tailed Student's t‐test (C,J–N). (^*^ *p* < 0.05, ^**^ *p* < 0.01, ^***^ *p* < 0.001, ^****^ *p* < 0.0001).

To identify m6A regulators that are most relevant to TNBC progression, we analyzed the correlation of the 10 major m6A modulators with patient survival. We found YTHDC1 and FTO were highly prognostic in TNBC, with elevated levels associated with reduced overall survival (OS) and progression‐free survival (PFS) in Kaplan–Meier plotter datasets (Figure 1A,B; Figures S1A,B). Clinical Proteomic Tumor Analysis Consortium (CPTAC) showed that YTHDC1, but not FTO, expressed higher in breast tumors than in adjacent normal tissues (Figure S1C), suggesting a more prominent role in oncogenesis. YTHDC1 expression was elevated in TNBC compared to other subtypes, and its high level significantly correlated with worse OS in TNBC, but not in ER^+^ and HER2^+^ subtypes (Figure S1D,E). Thus, we selected YTHDC1 for further study.

To validate YTHDC1 expression, we performed immunohistochemistry (IHC) on the tissue microarray (TMA) of breast cancer. YTHDC1 protein expression was significantly elevated in TNBC tissues compared with normal breast tissue and non‐TNBC subtypes (Figure 1C,D). Importantly, patients with high YTHDC1 staining in tumors had significantly shorter OS, as well as advanced tumor size, stage, and grade (Figure S2A and Table S1). YTHDC1 overexpression was also suggestive of poorer OS in the TNBC subtype in TMA (Figure S2A and Table S1), yet the finding did not reach statistical significance, which might be attributable to the limited sample size. In addition, YTHDC1 overexpression also tended to be associated with worse OS in the TCGA breast cancer dataset (Figure S2B), albeit not statistically significant. Together, these data suggest that YTHDC1 is a critical m6A modulator overexpressed in TNBC.

To explore the mechanism of how YTHDC1 regulated TNBC cells, we performed RNA‐sequencing (RNA‐seq) in MDA‐MB‐231 and Hs578T cells, two cell lines with highest YTHDC1 expression (Figure S2C), with siNC and siYTHDC1 (Figure S2D,E), as well as methylated RNA m6A immunoprecipitation sequencing (MeRIP‐seq/m6A‐seq) and YTHDC1 RNA immunoprecipitation sequencing (RIP‐seq) in MDA‐MB‐231 cells. Compared with the input, YTHDC1 RIP‐seq peaks were preferentially located at CDS loci (Figure S2F). The mRNA level of 1867 protein‐coding genes was significantly changed after YTHDC1 knockdown in both MDA‐MB‐231 and Hs578T cells (Figure S2G), among which 347 transcripts were both m6A methylated and bound to YTHDC1, and thus represented potential m6A‐dependent targets of YTHDC1 (Figure 1E). Gene ontology (GO) analysis revealed that these candidate genes were enriched in cell metabolic process (Figure 1F). METABRIC breast cancer data analysis also confirmed that YTHDC1 expression positively correlated with multiple metabolic signatures in TNBC cases, including glutamine (Gln) metabolism and the pentose phosphate pathway (PPP) (Figure 1G), with consistent findings observed across all breast cancer subtypes (Figure S2H). In the TCGA cohort, YTHDC1 expression correlated positively, though not significantly, with glutamate (Glu) signatures in TNBC (Figure S2I), whereas a significant correlation was observed across all breast cancer subtypes (Figure S2J).

We therefore analyzed whether YTHDC1 impacted metabolite profiles by mass spectrometry (MS). Glutamate metabolism, citrate cycle, glycolysis, and pentose phosphate pathway were found affected by YTHDC1 knockdown in MDA‐MB‐231 cells (Figure 1H), with most of the metabolites in these pathways being reduced after YTHDC1 knockdown (Figure 1I). Thus, we evaluated the effects of YTHDC1 on glucose uptake and glutamine metabolism (Figure 1J). Intracellular levels of G6P, Gln, Glu, and ATP decreased (Figure 1K–M; Figure S2K), but Gln uptake and Glu/Gln ratio increased in YTHDC1 knockdown cells (Figure S2L,M). Notably, we observed a decrease of glucose uptake in TNBC cells upon YTHDC1 knockdown (Figure 1N). Moreover, knocking down YTHDC1 remarkably inhibited cell proliferation and colony formation, while inducing cell death in TNBC cells (Figure S2N,O). Together, these results suggest that by rewiring glucose and glutamate metabolism, YTHDC1 drives tumor cell growth and dictates poor clinical prognosis of TNBC patients.

### 2.2 YTHDC1 Regulates ATF4 and GLUT3 mRNA Stability in an m6A‐Dependent Manner

**FIGURE 2:**
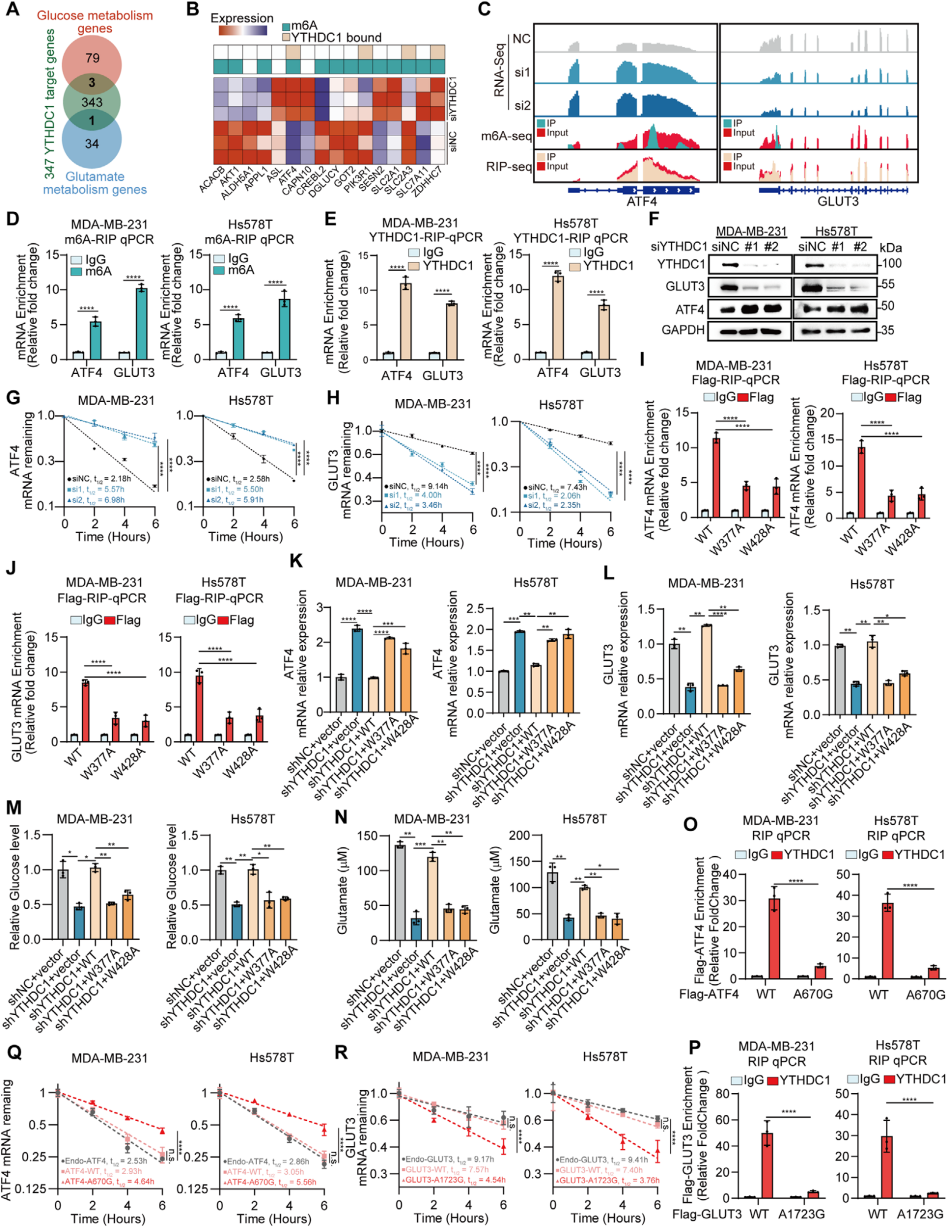
YTHDC1 regulates ATF4 and GLUT3 mRNA in an m6A‐dependent manner to coordinate TNBC metabolism. (A,B) Overlap of YTHDC1 target genes with genes in the glucose and glutamine pathways. (C) Representative tracks of RNA‐seq, m6A RIP‐seq, and YTHDC1 RIP‐seq at the ATF4 and GLUT3 loci in control versus YTHDC1 KD MDA‐MB‐231 cells, demonstrating YTHDC1 binding, m6A modification, and transcript abundance changes. (D) m6A RIP followed by RT‐qPCR showing m6A enrichment on ATF4 and GLUT3 transcripts in TNBC cells. (E) YTHDC1 RIP followed by RT‐qPCR in TNBC cells validates the binding of ATF4 and GLUT3 mRNAs to YTHDC1. (F) Western blot analysis of ATF4 and GLUT3 protein levels in TNBC cells upon YTHDC1 KD. (G) YTHDC1 KD increases ATF4 mRNA stability. (H) YTHDC1 KD decreases the stability of GLUT3 mRNA. (I,J) RIP‐qPCR in TNBC cells overexpressing Flag‐tagged WT or mutant YTHDC1 shows that YTHDC1 W377A and W428A mutations abolish the binding to ATF4 (I) and GLUT3 (J) mRNAs. (K,L) WT YTHDC1, but not the W377A and W428A mutants rescue the mRNA expression of ATF4 (K) and GLUT3 (L) in YTHDC1‐KD cells. (M,N) Intracellular glucose (M) and glutamate (N) levels in YTHDC1‐KD TNBC cells rescued with WT, but not mutant YTHDC1. (O,P) RIP assay showing reduced binding of the ATF4 A670G mutant transcript (O) and GLUT3 A1723G mutant transcript (P) to YTHDC1 compared to WT transcript. (Q,R) mRNA stability analysis demonstrating significantly enhanced stability of the A670G mutant compared to WT ATF4 (Q) and decreased stability of the A1723G mutant compared to WT GLUT3. Data are presented as mean ± SD of experimental triplicates (D,E,G–R). *p* values were determined using two‐tailed Student's t‐test. (*p < 0.05, ^**^ *p* < 0.01, ^***^ *p* < 0.001, ^****^ *p* < 0.0001, n.s means not significant).

Among genes involved in the glutamate metabolic pathway, only ATF4 was identified as a YTHDC1 target, while in the glucose metabolic pathway, GLUT3 (SLC2A3), PIK3R1, and ZDHHC7 were identified as YTHDC1 targets, with GLUT3 being associated with glucose transport (Figure 2A,B). These findings were consistently validated in both YTHDC1 RIP‐seq and MeRIP‐seq experiments (Figure 2A,B). As visualized by RNA‐seq tracks, ATF4 was upregulated whereas GLUT3 was downregulated following YTHDC1 knockdown in MDA‐MB‐231 cells (Figure 2C). Tracks from RIP‐seq and m6A‐seq further revealed enrichment of YTHDC1 binding and m6A methylation on ATF4 and GLUT3 mRNA, respectively (Figure 2C). To validate the m6A‐seq data, m6A‐RT‐qPCR was performed in TNBC cells, which confirmed significant m6A enrichment on ATF4 and GLUT3 mRNA relative to the IgG control (Figure 2D). Consistently, RIP assays demonstrated the binding of YTHDC1 to ATF4 and GLUT3 transcripts in both MDA‐MB‐231 and Hs578T cells (Figure 2E). YTHDC1 knockdown increased ATF4 expression while decreased GLUT3 expression in MDA‐MB‐231 and Hs578T cells (Figure 2F; Figure S3A). To further dissect how YTHDC1 regulates ATF4 and GLUT3 mRNA, we next examined the nuclear export, splicing, and stability of these transcripts. YTHDC1 knockdown did not affect the nuclear export and splicing of ATF4 and GLUT3 mRNA (Figure S3B–D), but lengthened ATF4 mRNA half‐life and shortened GLUT3 mRNA half‐life (Figure 2G,H).

To determine whether YTHDC1 exerts oncogenic functions depending on its m6A‐binding capacity, we constructed two known YTH m6A‐binding site mutants of YTHDC1 (W377A and W428A) (Figure S3E) [6]. RIP‐RT‐qPCR showed markedly decreased binding of ATF4 and GLUT3 mRNA to both Flag‐tagged mutants compared to wildtype (WT) YTHDC1 in MDA‐MB‐231 and Hs578T cells (Figure 2I,J). Overexpression of WT YTHDC1 and the mutants (W377A or W428A) were of comparable expression levels, but only WT YTHDC1 restored the expression and stability of ATF4 and GLUT3 mRNAs in MDA‐MB‐231 cells (Figure 2K,L; Figure S3F,G). Similar expression levels were confirmed in Hs578T cells. Consistent with these molecular changes, WT YTHDC1, but not the mutants, also rescued cell viability and colony formation in YTHDC1 knockdown MDA‐MB‐231 and Hs578T cells (Figure S3H,I). Moreover, in contrast to WT YTHDC1, neither the W377A nor W428A mutant could restore the impaired glucose, glutamate, and ATP levels in YTHDC1‐knockdown TNBC cells (Figure 2M,N; Figure S3J), demonstrating that this metabolic phenotype relies on the m6A‐binding function of YTHDC1.

To further establish that YTHDC1‐mediated metabolic regulation is dependent on m6A deposition rather than m6A‐independent RNA binding, we examined the epistatic relationship between m6A writers and YTHDC1. METTL3 was the major m6A writers for both ATF4 and GLUT3 transcripts, as predicted by RM2Target [11]. We overexpressed wild‐type YTHDC1 in METTL3 knockdown cells. Strikingly, unlike its efficient rescue in control cells, WT YTHDC1 completely failed to restore ATF4 and GLUT3 expression and metabolic phenotype in the METTL3 knockdown background (Figure S3K,L). SRAMP prediction [12] and our m6A‐seq data showed that c.A670 and c.A1723 were the m6A modification site on ATF4 and GLUT3 mRNA, respectively. Thus, we constructed the ATF4 mutant c.670A > G and GLUT3 mutant c.1723A > G (Tables S2 and S3 and Figure S3M–O). Compared to WT, the mutant showed significantly reduced binding abundance to YTHDC1 and markedly changed mRNA stability (Figure 2O–R; Figure S3P,Q). These data together provide evidence that YTHDC1 regulates cell metabolism in an m6A‐dependent manner.

### 2.3 YTHDC1 Knockdown Induces Disulfidptosis through ATF4‐Dependent SLC7A11 Upregulation

**FIGURE 3:**
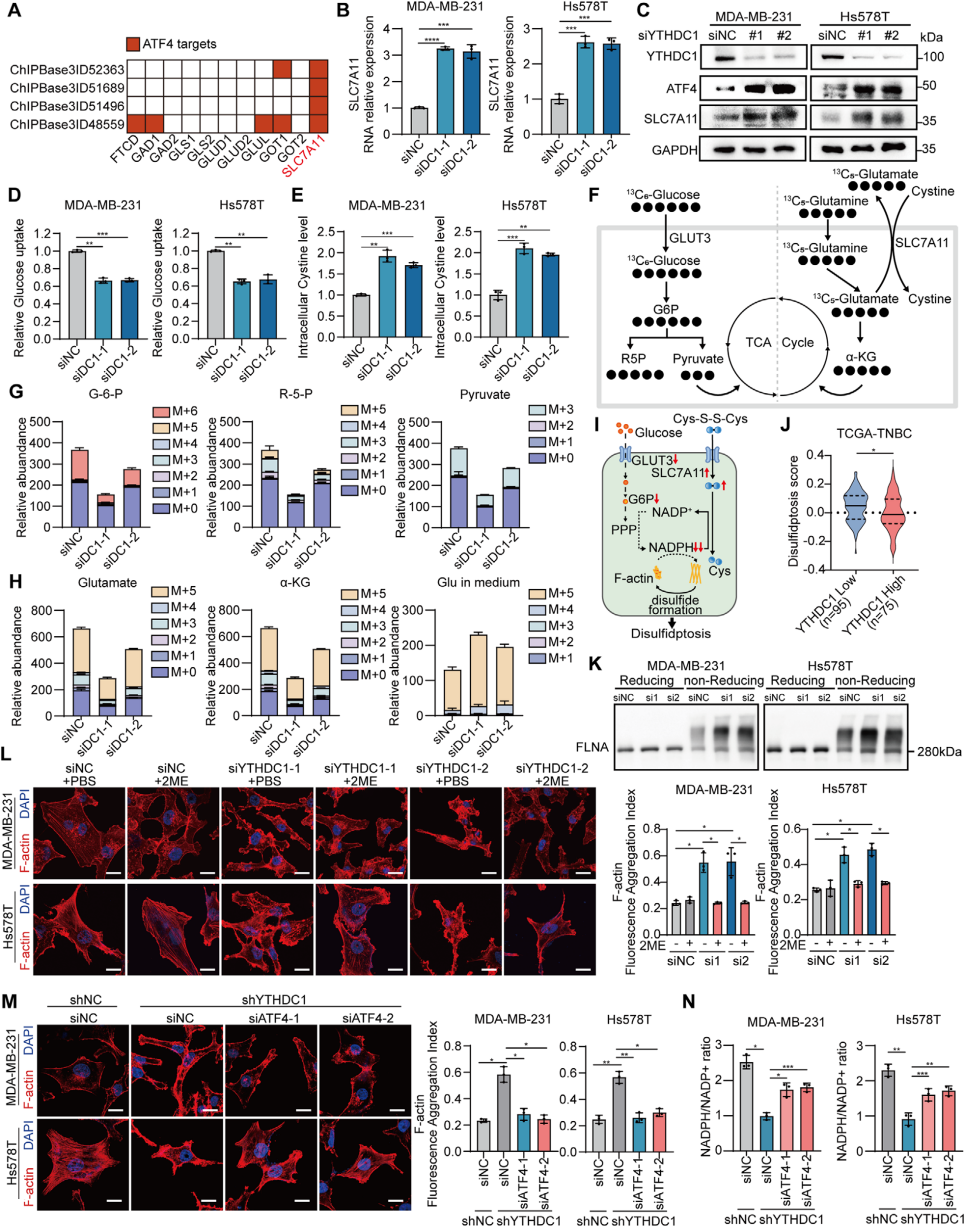
YTHDC1 knockdown induces disulfidptosis through ATF4‐mediated transcriptional upregulation of SLC7A11. (A) SLC7A11 is identified as a direct transcriptional target of ATF4 across multiple independent datasets in ChIPbase. (B,C) YTHDC1 knockdown in TNBC cells increases SLC7A11 expression at the (B) mRNA level and (C) protein level. (D,E) YTHDC1 knockdown decreases glucose uptake (D) and elevates intracellular cystine (E) in TNBC cells. (F) Schematic diagram of ^1^ ^3^C_5_‐glutamine or ^1^ ^3^C_6_‐glucose isotope tracing experiments followed by mass spectrometry in MDA‐MB‐231 cells. (G) YTHDC1 knockdown significantly reduced the abundance of ^1^ ^3^C‐labeled glucose‐6‐phosphate (G‐6‐P), ribose‐5‐phosphate (R‐5‐P), and pyruvate, compared with siNC cells. (H) YTHDC1 knockdown caused a marked reduction in intracellular ^1^ ^3^C‐labeled α‐ketoglutarate (α‐KG). Concomitantly, the abundance of ^1^ ^3^C‐labeled glutamate in the culture medium was significantly elevated. (I) A schematic diagram illustrating that upregulated SLC7A11 and downregulated GLUT3 together lead to disulfidptosis. (J) TNBC samples with low YTHDC1 expression exhibit higher disulfidptosis scores compared to those with high YTHDC1 expression in TCGA‐TNBC cohort. (K) Non‐reducing and reducing Western blotting show that YTHDC1 knockdown induces migration retardation of actin cytoskeleton proteins under non‐reducing conditions. (L) YTHDC1 knockdown induces disulfidptosis‐associated cell shrinkage and F‐actin contraction, which is rescued by 2‐mercaptoethanol (2‐ME) treatment. F‐actin is visualized by phalloidin staining, and F‐actin aggregation is quantified using the Fluorescence Aggregation Index (FAI). (M,N) F‐actin aggregation (M) and NADPH/NADP^+^ ratio (N) in YTHDC1‐KD TNBC cells are rescued with siATF4. Scale bars, 20 µm. Data are presented as mean ± SD of experimental triplicates (B,D,E, L–N). *p* values were determined using two‐tailed Student's t‐test. (^*^ *p* < 0.05, ^**^ *p* < 0.01, ^***^ *p* < 0.001, ^****^ *p* < 0.0001).

ATF4 has been reported to link cellular stress to glutamine metabolism [13]. To explore how ATF4 mediated the role of YTHDC1 in regulating glutamate metabolism, we searched downstream targets of ATF4 in ChIPbase [14]. Among key glutamate metabolism‐related genes, SLC7A11 was identified as an ATF4 target across four independent datasets (Figure 3A). Upon YTHDC1 knockdown in TNBC cells, the expression of SLC7A11 increased at mRNA and protein levels (Figure 3B,C). Immunofluorescence staining in TNBC cells also confirmed that YTHDC1 knockdown resulted in an elevated expression of SLC7A11 (Figure S4A). Moreover, YTHDC1 knockdown cells exhibited decreased intracellular glucose, elevated intracellular cystine, and reduced NADPH/NADP^+^ ratio (Figure 3D,E; Figure S4B).

To precisely define the metabolic flux changes orchestrated by YTHDC1, we performed isotope tracing experiments using ^1^ ^3^C_6_‐glucose and ^1^ ^3^C_5_‐glutamine coupled with mass spectrometry in YTHDC1‐knockdown and control TNBC cells (Figure 3F). ^1^ ^3^C_6_‐glucose tracing revealed that YTHDC1 knockdown caused a significant decrease in intracellular ^1^ ^3^C‐labeled glucose‐6‐phosphate (G6P), ribose‐5‐phosphate (R5P), and pyruvate (Figure 3G). These data directly confirm that YTHDC1 depletion suppresses glucose uptake and its subsequent channeling into glycolysis and the pentose phosphate pathway, consistent with YTHDC1‐mediated GLUT3 upregulation. For glutamine metabolism, ^1^ ^3^C_5_‐glutamine tracing showed that YTHDC1 knockdown led to a marked reduction in ^1^ ^3^C‐labeled α‐ketoglutarate (α‐KG) (Figure 3H). Concomitant with this intracellular depletion, the abundance of ^1^ ^3^C‐labeled glutamate in the culture medium was significantly elevated (Figure 3H), suggesting a glutamate efflux mechanism.

Simultaneous high expression of SLC7A11 and decreased glucose uptake are the primary triggers for disulfidptosis (Figure 3I) [15]. As YTHDC1 knockdown resembles the condition of SLC7A11 upregulation and reduced glucose uptake, we analyze whether YTHDC1 is associated with disulfidptosis in clinical samples using a previously reported algorithm [16]. In TCGA TNBC dataset, tumors with low YTHDC1 expression had significantly higher disulfidptosis scores compared to those with high YTHDC1 expression (Figure 3J). Thus, we further validated the impact of YTHDC1 on disulfidptosis phenotypes in MDA‐MB‐231 and Hs578T cells. Aberrant disulfide bonding in actin cytoskeleton proteins is a hallmark of disulfidptosis. We found that YTHDC1 knockdown in TNBC cells resulted in migration retardation of actin cytoskeleton proteins under non‐reducing conditions (Figure 3K). Phalloidin staining visualizing F‐actin revealed that YTHDC1 knockdown induced typical characteristic of disulfidptosis, including cell shrinkage and F‐actin contraction. These morphological alterations were reversed by 2‐mercaptoethanol (2‐ME) treatment (Figure 3L), a reducing reagent that could disrupt the disulfide bonds on F‐actin [16]. Importantly, all these phenotypes could be at least partially rescued by ATF4 knockdown (Figure 3M,N; Figure S4C–E). These data show that YTHDC1 knockdown induces disulfidptosis through ATF4‐dependent SLC7A11 upregulation.

To determine whether YTHDC1‐regulated glucose rewiring and glutamate rewiring represent independent parallel pathways or a compensatory network, first we cultured TNBC cells in glutamine‐deficient medium. YTHDC1 knockdown under glutamine deprivation further reduced intracellular glutamate levels and cell viability compared with glutamine deprivation alone. However, it did not significantly alter glucose uptake (Figure S4F‐H). As a parallel, to test whether glucose deprivation triggers compensatory glutamate utilization via YTHDC1, we subjected YTHDC1‐knockdown cells to glucose‐deficient conditions. While YTHDC1 knockdown under glucose deprivation further exacerbated glucose and ATP depletion, it did not significantly alter intracellular glutamate levels (Figure S4I–K). Collectively, these metabolic deprivation experiments reveal that YTHDC1‐regulated glucose rewiring and glutamate rewiring function as independent parallel pathways rather than hierarchically organized or compensatory networks.

To preliminarily explore the potential clinical utility of combining inhibition of these YTHDC1‐regulated metabolic axes with chemotherapy, we tested pharmacological inhibitors targeting the two pathways with taxane‐ or platinum‐based regimens, which were foundational chemotherapeutic agents for TNBC treatment. The GLUT1/3 inhibitor BAY‐876 combined with paclitaxel (PTX) exhibited markedly enhanced anti‐proliferative and pro‐apoptotic effects compared with either monotherapy (Figure S4L). Similarly, the glutaminase inhibitor CB‐839 combined with cisplatin (Cis) demonstrated significantly superior efficacy over single‐agent treatment (Figure S4M). Together, these data demonstrate that the independent parallel metabolic pathways orchestrated by YTHDC1 represent therapeutic vulnerabilities in TNBC and can be synergized with chemotherapy to enhance therapeutic efficacy.

### 2.4 YTHDC1 Drives Metabolic Rewiring and Confers Survival Advantages on TNBC Under Metabolic Stress In Vivo

**FIGURE 4:**
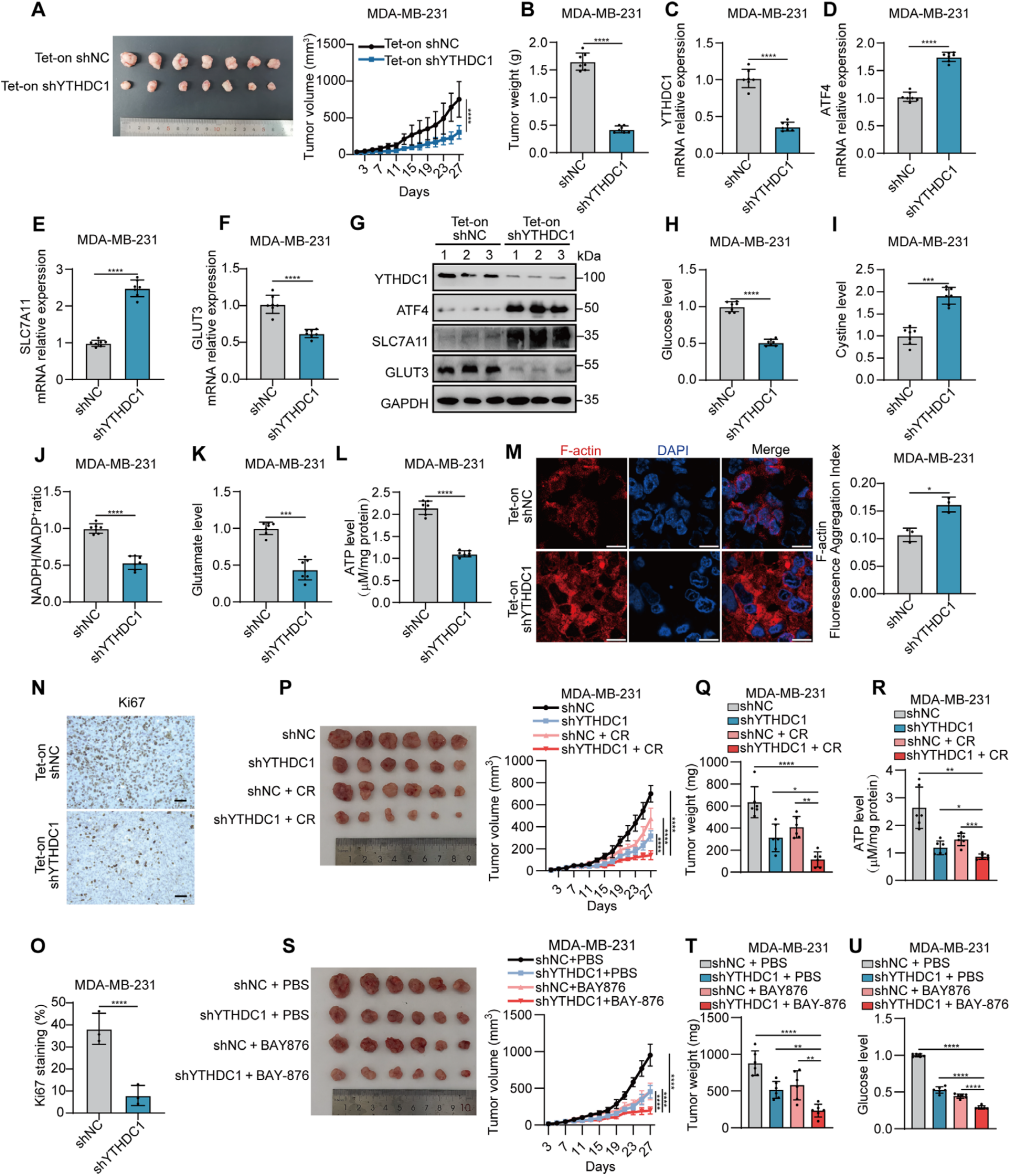
YTHDC1 knockdown suppresses tumor growth and induces disulfidptosis in vivo. (A,B) YTHDC1 knockdown suppresses the growth of MDA‐MB‐231 xenografts in nude mice. 2 × 106 MDA‐MB‐231 cells stably expressing Tet‐on control or YTHDC1 shRNA were injected as xenografts in nude mice. Doxycycline (200 µg/ml) was supplied in drinking water to induce Tet‐on shRNA expression. (C) Validation of YTHDC1 expression in xenograft tumors by RT‐qPCR. (D–G) YTHDC1 knockdown upregulates ATF4 and SLC7A11, and downregulates GLUT3 at the mRNA (D–F) and protein level (G) in xenograft tumors. (H–J) YTHDC1 knockdown leads to decreased intracellular glucose (H), elevated cystine (I), and a reduced NADPH/NADP+ ratio (J). (K,L) Measurement of intracellular glutamate (K) and ATP (L) levels in tumor tissues, showing significant depletion upon YTHDC1 knockdown. (M) Representative phalloidin staining shows increased F‐actin contraction and aggregation upon YTHDC1 knockdow, as quantified by a significant increase in the Fluorescence Aggregation Index (FAI). Scale bars, 10 µm. (N,O) Immunohistochemistry of Ki67 in in YTHDC1 knockdown and control tumor tissues. Scale bars, 50 µm. (P–R) 30% Caloric restriction (CR) combined with YTHDC1 knockdown remarkably inhibits tumor growth of MDA‐MB‐231 xenografts in nude mice. Tumor image and tumor growth curves (P), tumor weights (Q) and intratumoral ATP levels (R). (S–U) Pharmacological glucose restriction using BAY‐876 combined with YTHDC1 knockdown dramatically suppresses tumor growth of MDA‐MB‐231 xenografts in nude mice. Tumor image and tumor growth curves (S), tumor weights (T) and intratumoral glucose levels (U). For G, K, N, O, n = 3 mice per group. For AF, HJ, L, M, n = 7 mice per group. For PU, n = 6 mice per group. Each dot represents one mouse (A–U). Data are presented as mean ± SD (A–O, Q–R, T–U). For P and S, mean ± SEM are shown, and p values were determined using two‐tailed Student's t‐test. (*p < 0.05, **p < 0.01, ***p < 0.001, ****p < 0.0001).

To further validate our in vitro findings, we established a xenograft mouse model. MDA‐MB‐231 cells expressing Tet‐On shNC or shYTHDC1 were orthotopically inoculated into the mammary fat pads of nude mice. Upon doxycycline‐induced knockdown of YTHDC1, xenograft growth was significantly suppressed, as evidenced by the reduced volumes and weights of the tumors (Figure 4A,B). In YTHDC1 knockdown xenograft tumors, ATF4 and SLC7A11 expression were elevated at both the RNA and protein levels, while GLUT3 expression was reduced (Figure 4C–G). Concomitantly, these tumors exhibited decreased intracellular glucose, elevated cystine, and reduced NADPH/NADP^+^ ratio (Figure 4H–J). Moreover, Glu and ATP assay analysis revealed a marked decrease in glutamate and ATP (Figure 4K,L). Notably, F‐actin contraction was significantly increased in YTHDC1 knockdown tumors (Figure 4M), accompanied by reduced levels of Ki67 expression in YTHDC1 knockdown tumors (Figure 4N,O). By contrast, while YTHDC1 overexpression in BT549 cells also promoted xenograft tumor growth in nude mice, its effect was less pronounced than that of knockdown (Figure S5). These data suggest YTHDC1 deficiency elicits disulfide stress, creating a targetable vulnerability in TNBC that can be therapeutically exploited.

To further model metabolic stress in vivo, we subjected MDA‐MB‐231 xenograft‐bearing mice to 30% caloric restriction (CR) combined with doxycycline‐induced YTHDC1 knockdown. CR alone exerted a modest anti‐tumor effect, whereas YTHDC1 knockdown alone significantly suppressed tumor growth. Strikingly, the combination of CR and YTHDC1 knockdown resulted in profound tumor growth inhibition (Figure 4P,Q). Metabolic profiling of tumor tissues revealed that CR alone reduced intratumoral ATP levels (Figure 4R). CR combined with YTHDC1 knockdown caused catastrophic ATP depletion, indicating that YTHDC1‐deficient tumors lose the metabolic plasticity required to adapt to nutrient restriction.

To specifically model glucose‐deprivation stress while avoiding the systemic complexities of CR, we treated xenograft‐bearing mice with BAY‐876, a potent GLUT1/3 inhibitor, combined with YTHDC1 knockdown. BAY‐876 monotherapy moderately reduced tumor growth by imposing glucose restriction, whereas YTHDC1 knockdown alone suppressed tumor progression by disrupting metabolic rewiring (Figure 4S,T). Importantly, the combination treatment resulted in synergistic tumor suppression and near‐complete ablation of intratumoral glutamate pools (Figure 4U). Collectively, these in vivo metabolic stress models demonstrate that YTHDC1 is indispensable for TNBC cells to overcome metabolic stress.

### 2.5 ATF4 mRNA Delivery Effectively Reprograms the Metabolism of TNBC Cells

**FIGURE 5:**
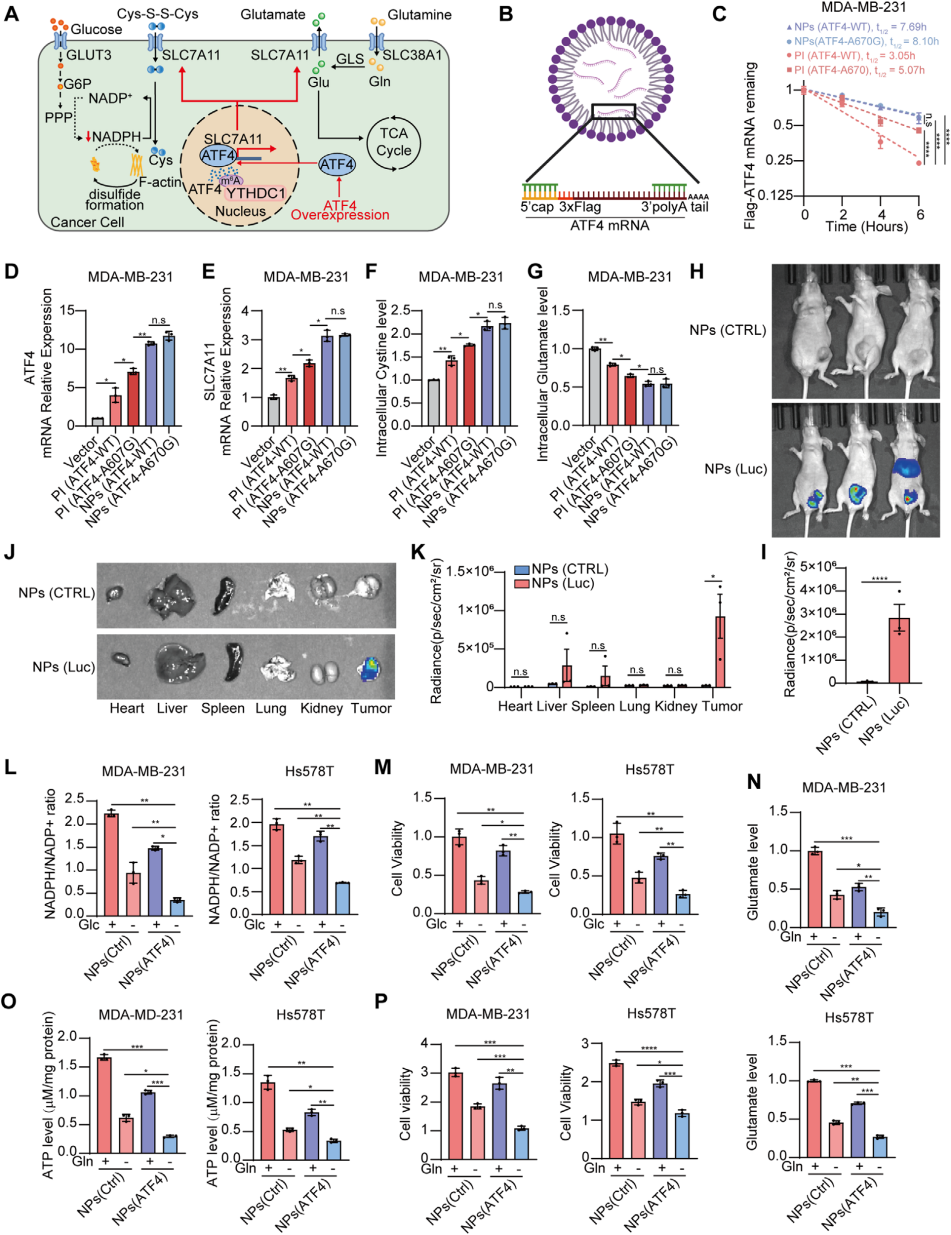
ATF4 mRNA nanoparticle delivery effectively reprograms the metabolism of TNBC cells. (A) The working model indicates that ATF4 overexpression enhances SLC7A11‐mediated cystine uptake and glutamate export, thereby sensitizing triple‐negative breast cancer cells to glutamine and glucose deprivation. B, Schematic illustration of the preparation of lipid‐polymer hybrid nanoparticles encapsulating ATF4 mRNA [NPs (ATF4‐WT)]. (C) NPs delivered ATF4 mRNA is more stable than plasmid‐transfected Flag‐ATF4 mRNA (WT and A670G). (D–G) NPs (ATF4‐WT) show higher ATF4 delivery efficiency (D), SLC7A11 expression (E), cystine uptake (F), and glutamate export (G) than plasmid‐based approaches, while the NPs (ATF4‐A670G) provide no additional advantage over NPs (ATF4‐WT). (H) Representative in vivo bioluminescence images of live mice bearing MDA‐MB‐231 xenografts following intratumoral injection of mFLuc‐loaded nanoparticles. (I) Quantification of total bioluminescence flux in tumors from the live mice shown in (H). (J) Representative bioluminescence images of tumors and major organs (heart, liver, spleen, lung, and kidney). (K) Quantitative analysis of bioluminescence signals in individual dissected organs and tumors shown in (J). (L,M) NPs (ATF4) further deplete NADPH (L) and impair cell viability (M) under glucose starvation. (N–P) NPs (ATF4) further deplete glutamate (N), reduce ATP (O), and impair cell viability (P) under glutamine starvation. Data are presented as mean ± SD of experimental triplicates (D–G,L–P). For (H–K), n = 3 mice per group. *p* values were determined using two‐tailed Student's *t*‐test (^*^ *p* < 0.05, ^**^ *p* < 0.01, ^***^ *p* < 0.001, ^****^ *p* < 0.0001, n.s means not significant).

Next, we sought to target the YTHDC1 pathway to reprogram tumor cell metabolism for TNBC intervention. Considering the critical roles of YTHDC1 in normal cells, we selected more specific downstream targets to minimize the potential toxicity. We focused on ATF4 because therapeutic overexpression of ATF4 offered combination strategies, enabling flexible treatment regimens with pharmacological agents. First, we hypothesized that ATF4 overexpression, which upregulated SLC7A11 and enhanced cystine import, might be combined with a GLUT inhibitor to induce disulfidptosis in TNBC. Second, ATF4 overexpression‐induced SLC7A11 mediates glutamate export, a crucial source to fuel the TCA cycle in TNBC cells (Figure 5A). We therefore hypothesized that ATF4 overexpression, combined with GLS inhibitor, which blocked the conversion of glutamine to glutamate, would synergistically deprive intracellular glutamate essential for cancer cell growth. Thus, we aim to develop a therapeutic overexpression system of ATF4.

To address the limitation of low delivery efficiency of plasmid transfection and circumvent YTHDC1‐mediated regulation, we synthesized ATF4 mRNA and encapsulated into P6CIT lipopolymer nanoparticles (LPNPs), an optimal mRNA delivery system [17], to form NPs (ATF4) (Figure 5B). Initially, we performed characterization tests on the synthesized nanoparticles (Figure S6A–F). Then we evaluated the expression efficiency of mRNA‐encapsulated nanoparticles in MDA‐MB‐231 cells. Nanoparticles loaded with ATF4 mRNA exhibited much higher overexpression efficiency than conventional liposome‐based plasmid transfection, and even higher than the mutant plasmid of ATF4 A670G, which was resistant to YTHDC1 recognition. As direct cytoplasmic delivery of ATF4 mRNA already enabled it to evade the nuclear function of YTHDC1, there was no significant difference of overexpression efficiency or mRNA stability between nanoparticles loaded with ATF4 mRNA and A670G mutant mRNA (Figure 5C,D). Specifically, treatment with nanoparticles encapsulating ATF4 mRNA at 1 µg/mL induced a more than 3‐fold increase in SLC7A11 expression, and significantly enhanced cystine level and decreased glutamate level in MDA‐MB‐231 cells (Figure 5E–G). These data suggest that the WT ATF4 mRNA delivered directly to the cytoplasm by nanoparticles effectively upregulates ATF4 expression and reprograms the metabolism of TNBC cells.

To test the efficiency of nano delivery in vivo, FLuc was encapsulated into Nano delivery via intratumoral injection into nude mice bearing MDA‐MB‐231 xenografts. In vivo imaging showed higher gene expression in tumor comparing to other major organs (Figure 5H–K). Moreover, treatment with NPs (ATF4) caused no significant impact on the major organs of the mice (Figure S6G).

Furthermore, we tested the effect of NPs (ATF4) in triple‐negative breast cancer cells for the combination strategies. First, under glucose deprivation, the use of NPs (ATF4) significantly reduced intracellular NADPH levels and suppressed cell viability in MDA‐MB‐231 and Hs578T cells (Figure 5L,M). Second, under glutamine deprivation, the use of NPs (ATF4) further decreased intracellular Glu and ATP levels (Figure 5N,O) in triple‐negative breast cancer cells and also suppressed cell viability (Figure 5P). These results suggest that the application of NPs (ATF4) sensitizes triple‐negative breast cancer cells to both glucose and glutamine deprivation.

### 2.6 Therapeutic Overexpression of ATF4 Combined with GLUT1 Inhibitor Induces Disulfidptosis in TNBC

**FIGURE 6:**
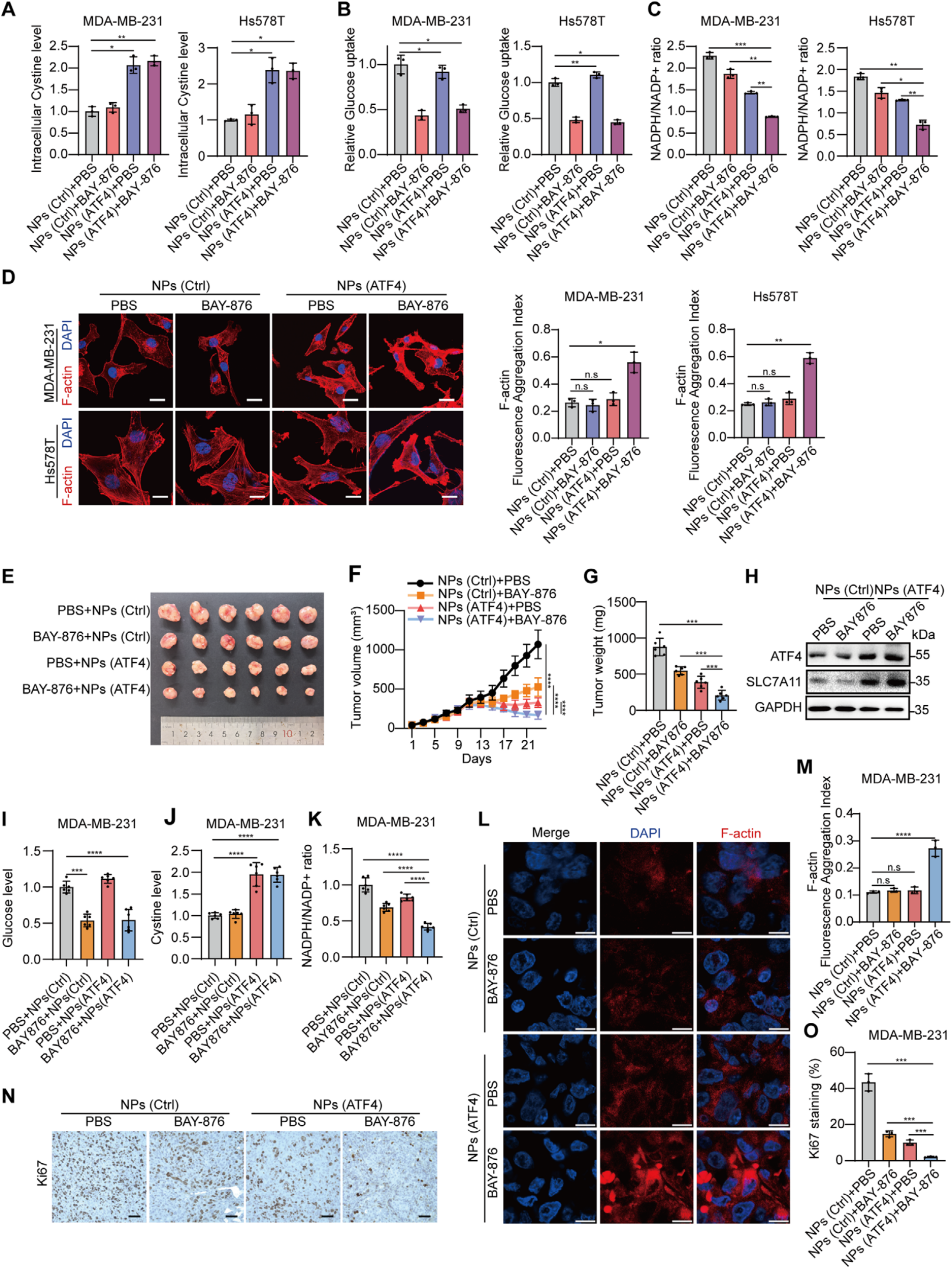
NPs (ATF4) combined with GLUTs inhibitor BAY‐876 synergistically induce disulfidptosis in vitro and in vivo. (A,B) Quantification of intracellular cystine levels (B) and glucose levels (C) in TNBC cells treated with PBS, NPs (ATF4), BAY‐876, or combination therapy. (C) NPs (ATF4) combined with BAY‐876 dramatically reduce NADPH/NADP^+^ ratio. (D) Representative phalloidin staining shows increased F‐actin contraction and aggregation upon combination treatment, which is quantified by a significant increase in the Fluorescence Aggregation Index (FAI). Scale bars, 20 µm. (E) The images of MDA‐MB‐231 xenograft tumors in nude mice treated with PBS, NPs (ATF4), BAY‐876, or combination therapy. (F,G) The combination of NPs (ATF4) and BAY‐876 induces superior antitumor efficacy compared to monotherapies or PBS control. (H) Western blot showing enhanced ATF4 and SLC7A11 protein expression in the NPs (ATF4) treated tumors. (I–K) Combination treatment decreases intracellular glucose (I), elevates cystine levels (J), and reduces NADPH/NADP^+^ ratio (K). (L,M) Representative phalloidin staining shows increased F‐actin contraction and aggregation upon combination treatment (L), which is quantified by a significant increase in FAI (M). Scale bars, 10 µm. (N,O) Immunohistochemical analysis shows markedly reduced Ki67‐positive cells in the combination group. Scale bars, 50 µm. Data are presented as mean ± SD of experimental triplicates (A–D). For (E–G,I–K), n = 6 mice per group. For (L–O), n = 3 mice per group. Each dot represents one mouse (G,I–K,M,O). *p* values were determined using two‐tailed Student's *t*‐test (^*^ *p* < 0.05, ^**^ *p* < 0.01, ^***^ *p* < 0.001, ^****^ *p* < 0.0001, n.s means not significant).

We investigated whether combining the NPs (ATF4) with GLUT1 inhibitor BAY‐876 could induce disulfidptosis in TNBC cells. Notably, single‐agent GLUT1 blockade showed limited effectiveness in clinical cancer treatment due to challenges like cancer cell adaptation, necessitating combination strategies to improve outcomes. Compared to PBS or either treatment alone, the combination of NPs (ATF4) and the GLUTs inhibitor BAY‐876 significantly increased intracellular cystine levels, decreased glucose uptake, and reduced the NADPH/NADP^+^ ratio in MDA‐MB‐231 and Hs578T cells (Figure 6A–C). We also observed cell shrinkage and F‐actin contraction in TNBC cells following the combined treatment (Figure 6D). Moreover, the combination treatment markedly inhibited cell viability and colony formation compared to monotherapy (Figure S7A,B).

Next, we combined NPs (ATF4) and BAY‐876 for in vivo treatment of orthotopically implanted MDA‐MB‐231 in nude mice. The combination treatment demonstrated potent tumor suppression compared to either PBS or monotherapy (Figure 6E–G). Intracellular delivery of ATF4 mRNA nanoparticles significantly enhanced the expression of ATF4 and SLC7A11 proteins in vivo (Figure 6H). Xenograft tumors treated with the combination of NPs (ATF4) and BAY‐876 exhibited decreased intracellular glucose, elevated cystine, and a reduced NADPH/NADP^+^ ratio (Figure 6I–K). Furthermore, tissue section analysis revealed dramatic F‐actin contraction (Figure 6L,M), and a marked decrease in Ki67 expression (Figure 6N,O). Thus, this combination therapy effectively inhibits tumor growth of YTHDC1‐overexpressed TNBC, demonstrating improved potency than BAY‐876 alone.

### 2.7 Therapeutic Overexpression of ATF4 Combined with GLS Inhibitor Leads to Severe Intracellular Glutamate Deprivation in TNBC

**FIGURE 7:**
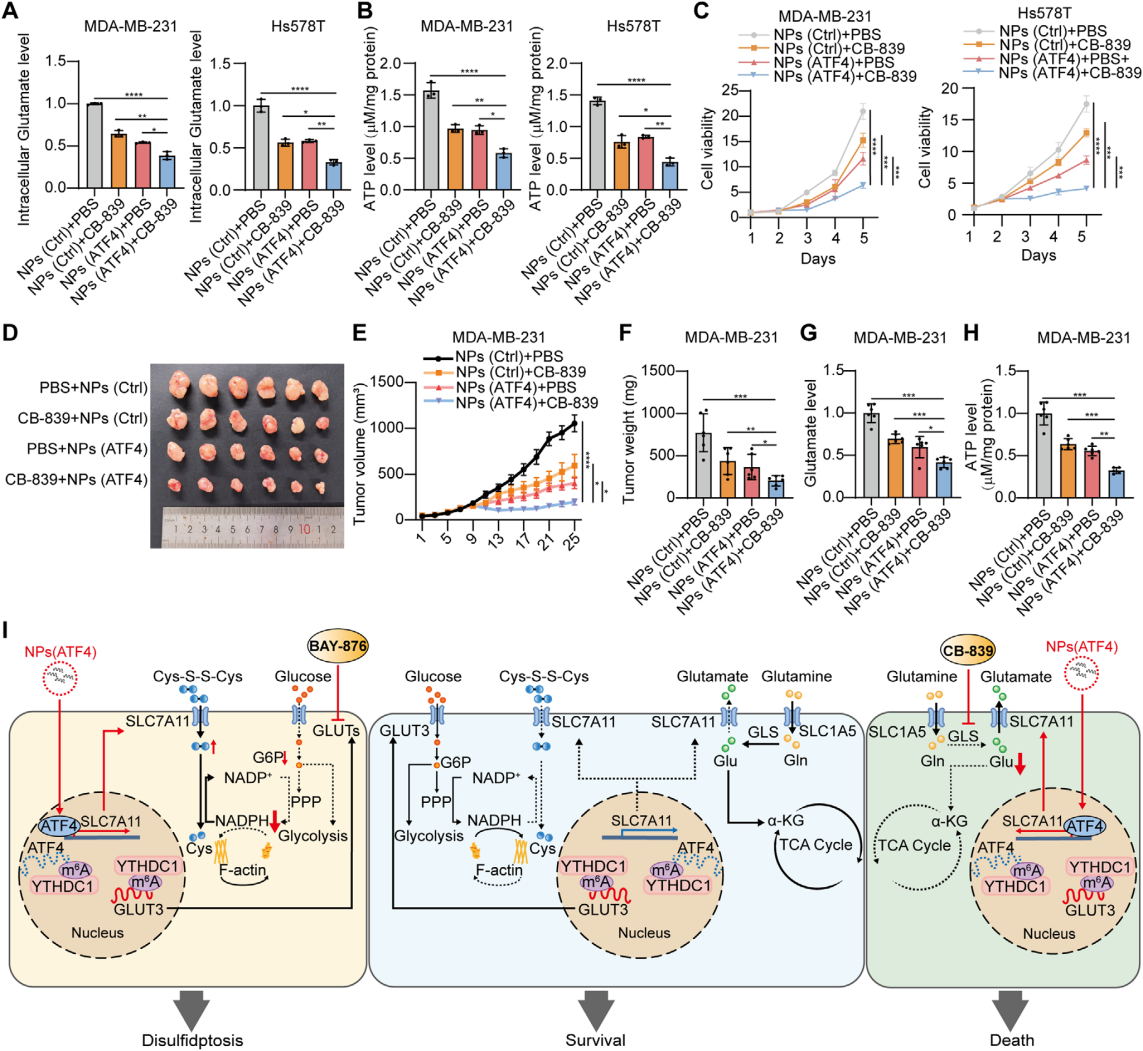
NPs (ATF4) combined with glutaminase inhibitor CB‐839 synergistically disrupts energy metabolism in vitro and in vivo. (A,B) Measurement of intracellular glutamate (A) and ATP (B) levels in TNBC cells treated with PBS, NPs (ATF4), CB‐839, or their combination. (C) Cell viability assessment reveals that the combination of NPs (ATF4) and CB‐839 exerts synergistic anti‐proliferative effects, significantly surpassing the efficacy of either monotherapy. (D) The images of MDA‐MB‐231 xenograft tumors in nude mice treated with PBS, NPs (ATF4), CB‐839, or combination therapy. (E,F) Growth curves and tumor weights show that the NPs (ATF4) and CB‐839 combination achieves superior tumor suppression compared to single‐agent treatments or PBS control. (G) Measurement of intratumoral glutamate levels demonstrates that combination treatment effectively depletes glutamate. (H) Measurement of ATP levels reveals that the combination therapy severely reduces cellular energy production in tumor tissues. (L) The working model illustrates that NPs (ATF4) enhance SLC7A11‐mediated cystine uptake and glutamate export in TNBC cells. When combined with GLUT1/3 inhibitor BAY‐876, glucose uptake and NADPH production are inhibited, which results in lethal disulfidptosis through redox imbalance. Another strategy is to combine with GLS inhibitor CB‐839, which further deprives intracellular glutamate, severely limiting energy production and leading to cell death. Data are presented as mean ± SD of experimental triplicates (A–C). For (D–H), n = 6 mice per group. Each dot represents one mouse (G,I–K,M,O). p values were determined using two‐tailed Student's t‐test (^*^ *p* < 0.05, ^**^ *p* < 0.01, ^***^ *p* < 0.001, ^****^ *p* < 0.0001).

Leveraging the role of YTHDC1 in regulating glutamine metabolism, we examined the therapeutic efficacy of combining NPs (ATF4) with a GLS inhibitor in TNBC. Of note, ATF4 overexpression‐induced SLC7A11 upregulation mediates the export of glutamate. To compensate for this loss and meet the high demand for intracellular glutamate, cancer cells become highly dependent on the conversion of glutamine to glutamate via GLS. Thus, we hypothesized that GLS inhibition could further deplete intracellular glutamate. We found that, compared to PBS or monotherapy, the CB‐839 and NPs (ATF4) combination more potently suppressed glutamate levels, ATP generation and cell viability in MDA‐MB‐231 and Hs578T cells (Figure 7A–C). The in vivo experiments showed that the NPs (ATF4) and CB‐839 combination significantly inhibited tumor growth and reduced glutamate and ATP levels relative to control or monotherapy groups, without observable weight loss (Figure 7D–H). Therefore, this combination therapy exploits a specific metabolic vulnerability in YTHDC1‐overexpressed TNBC, overcoming cellular adaptability, especially in glutamine‐addicted cancers.

Together, our study demonstrates that YTHDC1 orchestrates the metabolic reprogramming of TNBC cells by regulating the mRNA stability of ATF4 and GLUT3 in an m6A‐dependent manner, thereby sustaining metabolic homeostasis and conferring resistance to disulfidptosis. By delivering ATF4 mRNA directly to the cytoplasm and bypassing the nuclear m6A regulation of YTHDC1, NPs (ATF4) serves as a potent and versatile therapeutic agent, not only synergizing with GLUT inhibitors to induce disulfidptosis, but also cooperating with GLS inhibitors to trigger glutamate deprivation‐mediated cell death of TNBC cells (Figure 7I).

## 3 Discussion

Rather than acting on a single mRNA, m6A modulators target multiple transcripts to regulate their stability, translation, and splicing simultaneously. This mode of action enables m6A modulators to function as potential master regulators governing a specific cellular process. In this study, we revealed that the m6A reader YTHDC1, specifically associated with patient overall survival (OS) in the TNBC subtype, coordinated the rewiring of glucose and glutamate metabolism by regulating the mRNA stability of ATF4 and GLUT3, two key metabolic genes in TNBC. This glucose and glutamate rewiring is characterized by enhanced glucose uptake and channeling glucose carbon into glycolysis and the pentose phosphate pathway (PPP) to support NADPH production, while simultaneously preserving the intracellular glutamate pool through restrained glutamate export. Through this dynamic regulation, YTHDC1 endows TNBC cells with multiple growth advantages and enhances their resistance to diverse metabolic stresses. First, YTHDC1 stabilizes GLUT3 mRNA to sustain glucose uptake and NADPH production. Upregulated glucose uptake supports glycolysis and the pentose phosphate pathway for rapid biosynthesis and redox homeostasis. Second, YTHDC1 destabilizes ATF4 mRNA, leading to downregulated expression of SLC7A11 and consequently reducing cystine import and glutamate export. Under conditions of limited glucose availability, decreased influx of cystine alleviates NADPH depletion, thereby maintaining reductive capacity and protecting against disulfidptosis. Meanwhile, reduced glutamate export preserves the intracellular pool, allocating it for TCA cycle, glutathione production, and protein synthesis. Although YTHDC1 was reported to directly regulate SLC7A11 in neural cells [18], our RIP‐seq data did not detect significant YTHDC1–SLC7A11 mRNA interaction in TNBC cells, suggesting a cell‐type‐specific m6A regulation mode. Together, overexpression of YTHDC1 in TNBC drives m6A‐dependent metabolic reprogramming toward a state that fosters cell growth and enhanced adaptability, thereby conferring a survival advantage of TNBC cells.

Currently, a substantial number of drugs targeting cancer metabolism have been developed. Among the glucose transporter (GLUT) inhibitors, BAY‐876 (Bayer) demonstrates superior potency against GLUT1/3. Although the antitumor effects of BAY‐876 are well documented in various preclinical studies, its efficacy in TNBC models is constrained to pRB‐positive tumors or combined treatments with immunotherapy [19, 20]. Another class of therapeutics under active development are the inhibitors targeting glutamine and glutamate metabolism. The GLS inhibitor CB‐839 has undergone clinical trials in various tumor types with favorable outcomes. However, CB‐839 did not meet expectations in a phase I clinical trial for TNBC [21]. This may be attributed to that GLS is not a specific overexpressed target in TNBC cells, or its inhibition is readily compensated for by alternative pathways producing glutamate, thereby undermining monotherapy strategies.

Our data demonstrate that YTHDC1 specifically overexpresses in TNBC and correlates with unfavorable patient outcome. Targeting YTHDC1 not only suppresses GLUT3 expression and inhibits glucose uptake, but also upregulates SLC7A11, inducing disulfidptosis in TNBC cells. Meanwhile, YTHDC1 knockdown promotes glutamate export, further creating a therapeutic vulnerability of TNBC cells. Furthermore, in vivo experiments demonstrated that YTHDC1 confers survival advantage on TNBC subjected to caloric restriction or treated with the GLUTs inhibitor BAY‐876. However, it is crucial to note that m6A regulators play indispensable roles in normal physiological conditions [22]. YTHDC1 is essential for mRNA post‐transcriptional processing in normal cells [8, 23]. Therefore, targeting its downstream regulatory molecules offers a more viable approach to minimize adverse effects. Increasing evidence indicates that ATF4 agonists represent a potential strategy for cancer treatment [24, 25]. ATF4 agonist effectively suppressed the growth of neuroblastoma with dysregulated glutamine metabolism [26]. In the current study, we utilize a mRNA nanomedicine system to deliver ATF4 mRNA into cancer cells for therapeutic overexpression [17]. These nanoparticles exhibit significantly higher delivery efficiency than traditional plasmid transfection. A notable key difference of our system with common ATF4 agonist is that the delivered mRNA can evade the destabilization effect of YTHDC1, a nuclear protein, because the nanoparticles deliver mRNA directly to the cytoplasm, which does not enter the nucleus for m6A modification. Thus, exploiting the vulnerability conferred by YTHDC1 knockdown in TNBC, we devise a more specific alternative approach of delivering ATF4 mRNA, which bypasses m6A modification‐mediated regulatory mechanisms and achieves improved efficacy.

Based on our findings, we develop two ATF4 therapeutic strategies in this study. First, ATF4 overexpression in combination with the GLUT inhibitor BAY‐876 markedly reduced TNBC cell viability in vitro and in vivo. In the presence of BAY‐876‐induced glucose starvation, ATF4 overexpression upregulates SLC7A11 and enhances cystine import, triggering dramatic disulfidptosis in TNBC. Second, ATF4 overexpression combined with GLS inhibitor CB‐839 synergistically enhances glutamate efflux and reduces glutamine to glutamate conversion, which severely deprives intracellular glutamate and impairs cellular energy supply, leading to potent inhibition of TNBC tumor growth. However, these findings were derived from cell‐derived xenograft (CDX) models, which may not fully recapitulate the complexity of patient tumors. To facilitate clinical translation, these combination strategies using patient‐derived xenograft (PDX) models are needed in future studies. Collectively, these strategies effectively suppress tumor growth of TNBC models, and also offer combination regimens to improve the therapeutic efficacy of both GLUT inhibitors and GLS inhibitors whose utility have been constrained by modest single‐agent activity.

In summary, our study identifies YTHDC1 as a pivotal regulator that orchestrates glucose and glutamine metabolism to confer survival advantages to TNBC cells against lethal metabolic stress, especially disulfidptosis. Therapeutic overexpression of YTHDC1 downstream ATF4 by mRNA nanomedicine system in combination with GLUT or GLS inhibitors presents viable strategies to improve TNBC treatment outcomes, which also provides combination modalities for treating other cancers characterized by similar metabolic or signaling dependencies.

## 4 Experimental Section

### 4.1 Cell Culture

MDA‐MB‐231, Hs578T and BT549 TNBC cells were obtained from American Type Culture Collection (ATCC). MDA‐MB‐231 and Hs578T cells were cultured in DMEM (Gibco) supplemented with 10% fetal bovine serum (FBS, Vigonob) and 1% penicillin/streptomycin (Gibco). BT549 cells were cultured in RPMI‐1640 (Gibco) supplemented with 10% FBS and 1% penicillin/streptomycin.

### 4.2 Constructs, Cell Transfection, and Infection

The siRNA/shRNA sequences (RIBOBIO) are listed in Table S4. shRNA sequences were cloned into pLKO‐Tet‐On plasmid for lentivirus‐mediated knockdown. Flag‐tagged YTHDC1 WT, YTHDC1 W377A, and YTHDC1 W428A were cloned into pcDNA3.1 plasmids for ectopic expression. YTHDC1 W377A and W428A were generated by PCR‐based site‐directed mutagenesis, changing codon 377 and codon 428 from TGG (Trp) to GCG (Ala). Synonymous mutations without changing the resulting amino acid were introduced into the RNAi‐targeting site of both wild‐type and mutant YTHDC1 to prevent knockdown of the ectopically expressed protein, changing Ala‐Arg‐Ser‐Val‐Ile‐Leu from GCA‐AGG‐AGT‐GTT‐ATC‐TTA to GCC‐CGC‐AGC‐GTC‐ATT‐CTG. To generate plasmids encoding Flag ATF4 A670G (changing nucleotide 670 from A to G) and Flag GLUT3 A1723G (changing nucleotide 1723 from A to G), point mutations that substitute the adenosine within the m6A consensus motif (DRACH) with guanosine were introduced into wild‐type Flag‐ATF4 and Flag‐GLUT3 pcDNA3.1plasmids. The vector, YTHDC1, ATF4, and GLUT3 gene sequence were digested by KpnI and PacI restriction enzymes, and complete cloning through In‐fusion recombination method. All constructs were verified by Sanger sequencing.

Cells were transfected with indicated siRNAs using Lipofectamine RNAiMAX (Invitrogen, 13778150), or plasmids using Lipofectamine 3000 (Invitrogen, #L3000008). After transducing with lentiviral particles, the cells were selected with puromycin (1 µg/ml) to generate stable cell lines and treated with doxycycline (100 ng/ml) to induce Tet‐On shRNA expression for YTHDC1 knockdown.

### 4.3 RNA Immunoprecipitation (RIP)

RIP experiments were performed with Magna RIP RNA‐Binding Protein Immunoprecipitation Kit (Millipore, 17–700). Cell lysates were collected with IP lysis buffer (Thermo Fisher Scientific, 87787) containing 1% ribonuclease inhibitor (Promega, N2515), Protease and Phosphatase Inhibitor (Thermo Fisher Scientific, 78437), and EDTA (Thermo Fisher Scientific, 17892). The cells were lysed at 4°C for 30 min and centrifuged at 4°C at 12 000 g for 20 min. The supernatant was transferred into a new EP tube, and 5% of the volume was taken as input storing at −80°C. The lysates were incubated with primary antibody‐coupled magnetic beads for 4 h at 4°C. Anti‐Flag (Sigma, F3040) (1:200) and anti‐YTHDC1 (Abclonal, A22992) (1:100) for RIP, anti‐m6A (Abclonal, A19841) (1:50) for m6A‐RIP. Then the RNA in immunoprecipitated complex was extracted for sequencing or RT‐qPCR.

### 4.4 Metabolite Extraction and Mass‐Spectrometry‐Based Metabolomics Analysis

Cells were maintained in regular growth medium, and 1 mL of cold 80% methanol (LC/MS) was added for metabolite extraction. The supernatant was transferred into a new Eppendorf tube, while the pellet was resuspended in 1 mL of cold 80% methanol for a second round of extraction. The extracted metabolites were finally resuspended in 80% acetonitrile (ACN). The extracted metabolites were identified by Poroshell 120 EC‐C18 column (2.1 × 100 mm; 2.7 µm; Agilent, USA) and Agilent Infinity 1290II‐6495A LC‐MS system (Agilent Technologies, Inc., CA, USA). Data were normalized to the cell number. The enriched metabolic pathways of significantly changed metabolites with default parameters were analyzed by Metaboanalyst.

### 4.5 ^1^ ^3^C_5_‐Glutamine / ^1^ ^3^C_6_‐Glucose Tracing Followed by Mass Spectrometry

For ^1^ ^3^C_6_‐glucose tracing, MDA‐MB‐231 cells were seeded in 6‐well plates at a density of 1 × 10^6^ cells per well and cultured in glucose‐free DMEM (Servicebio, G4583) supplemented with 10% fetal bovine serum (FBS) and D‐Glucose‐^13^C_6_ (MCE, HY‐B0389A, 2 g/L final concentration) for 1 h. Unlabeled control cells were maintained in parallel with standard DMEM. 1 mL cold 80% methanol (LC/MS) was added to cells. The supernatant was transferred to a new Eppendorf tube, and pellet was resuspended in 1 mL cold 80% methanol for second extraction.

For ^1^ ^3^C_5_‐glutamine tracing, MDA‐MB‐231 cells were seeded in 6‐well plates at a density of 1 × 10^6^ cells per well and cultured in glutamine ‐free DMEM (Servicebio, G4517) supplemented with 10% fetal bovine serum (FBS), and L‐Glutamine‐^13^C_5_ (MCE, HY‐N0390S1, 4 mM final concentration) for 3 h. The cell culture medium was collected, centrifuged at 1000 × g for 5 min at 4°C to remove cell debris, and the supernatant was immediately flash‐frozen in liquid nitrogen and stored at −80°C until LC‐MS/MS analysis. 1 mL cold 80% methanol (LC/MS) was added to cells. The supernatant was transferred to a new Eppendorf tube, and pellet was resuspended in 1 mL cold 80% methanol for second extraction. The extracted metabolites were detected by Agilent 8890–7000D system (Agilent Technologies, USA).

### 4.6 RNA‐seq, m6A‐seq, and RIP‐seq

RNA‐seq was performed on siNC and siYTHDC1 TNBC cells (MDA‐MB‐231 and Hs578T). For m6A‐seq, fragmented RNA was immunoprecipitated with an anti‐m6A antibody in MDA‐MB‐231. For RIP‐seq, RNA was immunoprecipitated with an anti‐YTHDC1 antibody using normal IgG as the negative control. All libraries were subjected to paired‐end 150‐bp sequencing on an Illumina NovaSeq 6000 platform. Matched input controls were included for all m6A‐seq and RIP‐seq samples, MACS2 was run with the built‐in input normalization parameter. Different expressed genes (DEGs) in RNA‐seq were identified with |log_2_ fold change| > 1 and *p* value < 0.05. Differential m6A peaks and RIP‐seq peaks were called with FDR < 0.05, and direct targets of YTHDC1 were determined by integrating RIP‐seq and RNA‐seq data.

### 4.7 Analysis of Glutamate, Glutamine, and Cysteine

For glutamine measurement and glutamine uptake assay, the glutamine level was determined using a Glutamine Assay Kit (Abcam, ab197011) according to the manufacturer's instructions. Cells cultured in fresh complete medium were seeded into 6‐well plates. An aliquot of medium was reserved to determine the glutamine level at the initial time point (T0). After 24 h of incubation (T24), the culture supernatant was collected, and the glutamine level was measured. Glutamine uptake was then calculated by subtracting the glutamine concentration at T24 from that at T0. After removal of the supernatant, tumor cells were lysed, and intracellular glutamine content was quantified.

For glutamate measurement, glutamate release assay and Glu/Gln ratio assay, glutamate levels were determined using a Glutamate Assay Kit (Solarbio, BC‐1585) according to the manufacturer's instructions. Briefly, Cells cultured in fresh complete medium were seeded into a 96‐well plate. The medium was removed, and the cells were washed once with PBS, followed by incubation in PBS for 2 h. The supernatant was then collected to measure glutamate content, and glutamate release was calculated. After removing the supernatant, the tumor cells were lysed to determine the intracellular residual glutamate content and Glu/Gln ratio.

For cystine uptake assay, the uptake of cystine of cells was measured by Cystine Uptake Fluorometric Assay Kit (Elabscience, E‐BC‐F066) according to the manufacturer. Briefly, cells were cultured in fresh complete medium, 50 µL of cell lysate was aspirated and added to each well of microplates for fluorescence‐based assays for detection.

### 4.8 Glucose Uptake Assay

The uptake of glucose of cells was measured by Glucose Uptake Fluorescence Assay Kit with 2‐NBDG (Beyotime, S0561S) and Glucose Uptake Assay Kit with WST‐8 (Beyotime, S0554S) according to the manufacturer. For the Kit with 2‐NBDG, an appropriate volume of the 2‐NBDG glucose uptake working solution was added to the culture medium, followed by incubation at 37°C in a cell culture incubator for 30 min. After replacing with fresh normal culture medium, images were captured under a fluorescence microscope. For the Kit with WST‐8, Glucose Uptake Lysis Buffer was added to the culture dish, and the cells were gently pipetted to ensure thorough lysis. The lysate was centrifuged at 4°C and 14 000 × g for 5 min. The supernatant was then collected, mixed with Glucose Uptake Detection Reagent, and incubated at 37°C for 30 min. Finally, the absorbance was measured at 450 nm.

### 4.9 Intracellular Glucose‐6‐Phosphate Measurement

The intracellular levels of Glucose‐6‐phosphate was measured by G6P Assay Kit with WST‐8 (Beyotime, S0185) according to the manufacturer. The cells were lysed by adding G6P extraction solution, followed by the addition of G6P detection solution. The mixture was then incubated at 37°C for 10 min under light‐protected conditions, and the absorbance was measured at 450 nm.

### 4.10 Intracellular NADPH/NADP^+^ Ratio Measurement

The intracellular levels of NADPH and total NADP were measured by an NADP^+^/NADPH Assay Kit (Beyotime, S0179) according to the manufacturer. The concentration of NADP^+^ was calculated by subtracting NADPH from total NADP.

### 4.11 ATP Level Measurement

ATP levels were determined using the Enhanced ATP Assay Kit (Beyotime, S0027) according to the manufacturer's protocol. Briefly, ATP was released by cell lysis. Then, an appropriate amount of ATP test solution was added to the cell lysate. Lastly, the plate was read within 1–2 min on a Synergy H1 microplate reader (BioTek Instruments, Inc).

### 4.12 Cytoskeleton Staining

Cells were plated in confocal dishes and cultured overnight. Following washes with pre‐chilled PBS, the cells were fixed in 4% paraformaldehyde, permeabilized in 0.5% Triton X‐100, and blocked in PBST supplemented with 10% normal goat serum. The cytoskeleton was stained with Rhodamine‐conjugated phalloidin (Beyotime, C2207S; 1:200), while the nuclei were counterstained with DAPI. Images were captured on a Leica SP8 Lightning confocal microscope equipped with Leica LAS X 4.0 data acquisition software. Finally, we estimated the Fluorescence Aggregation Index (FAI) as the following formula: FAI = (Area2 × Mean2)/(Area1 × Mean1). Area1 is the total cell area, and Area2 the area of high fluorescence intensity region. Mean1 is the average fluorescence intensity, and Mean2 is the average fluorescence intensity in the high fluorescence intensity region. High fluorescence intensity region is defined as areas where fluorescence intensity > 2× Mean1.

### 4.13 Total RNA Extraction and Real‐Time qPCR (RT‐qPCR) Analysis

Total RNA was isolated using TRIzol reagent (GLPBIO, GK20008) following the manufacturer's instructions. Then 1000 ng of total RNA was reversely transcribed into first‐strand cDNA with the PrimeScript RT Master Mix kit (TAKARA, RR036A). qPCR was carried out using UNICON qPCR SYBR Green Master Mix (Yisheng, 11198ES08) on a LightCycler 480 system (Roche). Relative levels of target genes were calculated with the CT method and normalized to GAPDH or β‐actin. Primer sequences are listed in Table S5.

### 4.14 RNA Stability Assays

Cultured cells were treated with 5 mg/L actinomycin D (Selleck, S8964) and harvested at the indicated time points. Total RNA was then extracted with TRIzol reagent and subjected to RT‐qPCR analysis. The mRNA turnover rate and half‐life were estimated according to a previously published study [27].

### 4.15 Western Blot

Cells were lysed with RIPA buffer (Beyotime, P0013B) supplemented with 100× protease inhibitor cocktail (Thermo Fisher Scientific, 78437) at 4°C for 30 min. Equal amounts of protein were separated by 10% SDS‐PAGE and subsequently transferred onto PVDF membranes. After blocking with 5% non‐fat milk, the membranes were sequentially incubated with primary and secondary antibodies, and protein bands were visualized with ECL reagent. The following primary antibodies were used for western blot: anti‐YTHDC1 (ABclonal, A22992; 1:500), anti‐ATF4 (ABclonal, A0201; 1:1000), anti‐SLC7A11 (Abcam, ab307601; 1:1000), anti‐GLUT3 (ABclonal, A4137; 1:1000), anti‐FLNA (ABclonal, A3738; 1:2000), and anti‐GAPDH (ABclonal, A19056; 1:10000). For western blot under non‐reducing conditions, protein samples were mixed with SDS‐PAGE Protein Loading Buffer (MCE, HY‐K1108, non‐reducing, 5×) containing no reducing agents, and each sample was divided into two aliquots, one aliquot was subjected to non‐reducing analysis, whereas the other was supplemented with 2% (final concentration) β‐mercaptoethanol for reducing analysis.

### 4.16 Immunohistochemistry

The tissue microarray (TMA) of breast cancer tissues with clinicopathological information (Outdo Biotech, XT18‐015 and XT19‐001) were used for YTHDC1 immunohistochemical (IHC) analysis. Briefly, antigen retrieval was performed by citric acid buffer. YTHDC1 antibody (ABclonal, A22992) (1:500) was incubated overnight at 4°C, and secondary antibodies was incubated for 1 h at 37°C. Staining was carried out using the DAB peroxidase substrate kit (Vector Laboratories). IHC images were randomly captured at 40× magnification using an Olympus BX43 microscope. The staining index (SI) of YTHDC1 was calculated as the product of the staining intensity and the proportion of YTHDC1‐positive tumor cells, based on the evaluation of at least 5 randomly selected fields (objective ×40). Specifically, the staining intensity was graded on a four‐tier scale (0, no YTHDC1 staining; 1, weak; 2, moderate; 3, strong), while the proportion of YTHDC1‐positive tumor cells was categorized into four grades (1, <25% YTHDC1‐positive cells; 2, 25%–50%; 3, 50%–75%; 4, >75%). Consequently, YTHDC1 expression was quantified as an SI ranging from 0 to 12. An optimal SI cutoff value (<5, low; ≥5, high) was determined with X‐tile software.

### 4.17 Preparation of Nanoparticles

In vitro transcription (IVT) of ATF4 (NCBI Gene ID: 468) was performed using the Standard mRNA Synthesis kit (NEB, E2060s). Briefly, the plasmid harboring the ATF4 sequence was first linearized by BspQI digestion, and the linearized plasmid was then used as the template for IVT. The IVT reaction was employed following the manufacturer's protocol with 3‐OH AG (cap1) as the cap analog. The transcribed mRNAs were purified using VAHTS mRNA capture beads, and the final mRNA concentration was quantified with a NanoDrop spectrophotometer.

The NPs were synthesized as previously described [17]. The hydrodynamic size, polydispersity index (PDI), and zeta potential of the LPNPs were measured by dynamic light scattering (DLS, Malvern, UK), while their morphology was examined under transmission electron microscopy (TEM).

### 4.18 Animal Experiments

Three‐ to five‐week‐old SPF‐level BALB/C nude female mice weighing 14–16 g was purchased from Vital River Laboratory and housed in the barrier environment. All animal experiments were conducted in accordance with protocols approved by the Institutional Animal Care and Use Committee of Sun Yat‐sen Memorial Hospital (Approval Number AP20230277, AP20250131, AP20250277, and AP20260089). To assess the impact of YTHDC1 on xenograft tumor growth, 1 × 10^6^ MDA‐MB‐231 cells (shNC vs shYTHDC1) or 2 × 10^6^ BT549 (vector vs YTHDC1) were injected orthotopically into mammary fat pad of BALB/C nude mice. Induction of Tet‐on shRNA expression was achieved by supplementing the drinking water with doxycycline (200 µg/mL). For caloric restriction (CR) experiments, mice were randomized to receive 30% caloric restriction (relative to the average daily intake of the control group) while maintaining essential nutrient sufficiency [28]. For pharmacological glucose restriction, mice were treated with BAY‐876 (3 mg/kg body weight) or vehicle control by PBS twice daily for 7 consecutive days. Once tumors were palpable, tumor size was measured every 2 days, with tumor volume calculated according to the formula: volume = length × width × width/2.

To assess the tumor targeting ability and safety of the nano‐delivery system, 1 × 10^6^ MDA‐MB‐231 cells were orthotopically injected into the mammary fat pad of BALB/C nude mice. When tumors became palpable, tumor‐bearing mice were randomly divided into two groups and treated as follows: i) NPs (Ctrl), ii) NPs (Luc). The biodistribution of NPs was tracked by quantifying the average fluorescence intensity from bioluminescence imaging. Before imaging, mice received an intraperitoneal injection of D‐luciferin substrate, and signals were acquired using an IVIS Lumina III imaging system (PerkinElmer, USA). To evaluate the effect of NPs (ATF4) combined with BAY‐876 and CB‐839 on xenograft tumor growth, 1 × 10^6^ MDA‐MB‐231 cells were orthotopically injected into the mammary fat pad of BALB/C nude mice. When tumors became palpable, tumor‐bearing mice were randomly divided into four groups and treated every other day as follows: i) PBS + NPs (Ctrl), ii) PBS + NPs (ATF4), iii) BAY‐876 or CB‐839 + NPs (Ctrl), iv) BAY‐876 or CB‐839 + NPs (ATF4). A total of three treatment cycles were administered. NPs (ATF4) were given via intratumoral injection at a dose of 150 µg/kg, BAY‐876 was administered by oral gavage at 3 mg/kg, and CB‐839 was administered by oral gavage at 100 mg/kg. At the experimental endpoint, tumor tissues were harvested, photographed, and frozen at −80°C for subsequent RNA and protein extraction, or fixed with 4% paraformaldehyde for immunohistochemical analysis.

### 4.19 Statistical Analyses

All statistical analyses were conducted with R‐Studio. Two‐group comparisons were evaluated using the two‐sided Student's t test. For survival analysis, Kaplan–Meier survival curves were generated in R‐Studio. *p* value < 0.05 was considered statistically significant.

## Author Contributions

Conceptualization: **Zheng‐Hao Lai**, **Na‐Na Li**, and **Man‐Li Luo**. Methodology: **Zheng‐Hao Lai**, **Yi Wang**, **Na‐Na Li**, **Ling Zeng**, **Xue Li**, **Renzhong Liu**, **Yingsen Tang**, and **Jinjin Chen**. Investigation: **Zheng‐Hao Lai**, **Yi Wang**, **Na‐Na Li**, **Ling Zeng**, **Xue Li**, **Renzhong Liu**, **Xin‐Yu Ma**, **Cheukfai Li**, **Wenkui Fu**, and **Yingsen Tang**. Visualization: **Zheng‐Hao Lai**, **Ling Zeng**, **Yi Wang**, and **Xin‐Yu Ma**. Funding acquisition: **Ning Liao** and **Man‐Li Luo**. Project administration: **Man‐Li Luo**, **Ning Liao**, **Jinjin Chen**, and **Na‐Na Li**. Supervision: **Ning Liao** and **Man‐Li Luo**. Writing – original draft: **Zheng‐Hao Lai**. Writing – review & editing: **Man‐Li Luo**.

## Conflicts of Interest

The authors declare no conflicts of interest.

## Supporting information




**Supporting File**: advs77492‐sup‐0001‐SuppMat.pdf.

## Data Availability

The data that support the findings of this study are openly available in GEO at https://www.ncbi.nlm.nih.gov/geo/, reference number GSE317469, GSE318636.
