# Supplementary material for: YTHDC1 Orchestrates Glucose and Glutamate Rewiring to Overcome Lethal Metabolic Stress in Triple‐Negative Breast Cancer
Source: Adv Sci (Weinh). 2026 Aug 30:e77492. Online ahead of print. doi: 10.1002/advs.77492 (PMC13526702; doi:10.1002/advs.77492)
Supplement: Supplementary file 1 [file ADVS-9999-e77492-s001.pdf]

Supplementary Materials

YTHDC1 orchestrates glucose and glutamate rewiring to overcome lethal metabolic stress in triple-negative breast cancer

Zheng-Hao Lai[1,2] | Yi Wang[1,2] | Na-Na Li[1,2] | Ling Zeng[1,2] | Renzhong Liu[1,2] | Xue Li[1,3] | Cheukfai Li[4] | Xin-Yu Ma[1,2] | Wenkui Fu[1,2] | Yingsen Tang[1,2] | Jinjin Chen[1,2] | Ning Liao[4, *] | Man-Li Luo[1,2, *]

[1]Medical Research Center, Sun Yat-Sen Memorial Hospital, Sun Yat-Sen University, Guangzhou 510120, China. | [2]Guangdong Provincial Key Laboratory of Malignant Tumor Epigenetics and Gene Regulation, Sun Yat-Sen Memorial Hospital, Sun Yat-Sen University, Guangzhou 510120, China. | [3]Wuxi Hospital Affiliated to Nanjing University of Chinese Medicine, Wuxi 214071, China. | [4]Department of Breast Surgery, Guangdong Provincial People's Hospital and Guangdong Academy of Medical Sciences, Guangzhou 510080, China.

* **Correspondence:** Ning Liao (syliaoning@scut.edu.cn) | Man-Li Luo (luomli@mail.sysu.edu.cn)

Supplementary Materials includes:

Supplementary Figures 1-7

Supplementary Tables 1-5

Figure S1

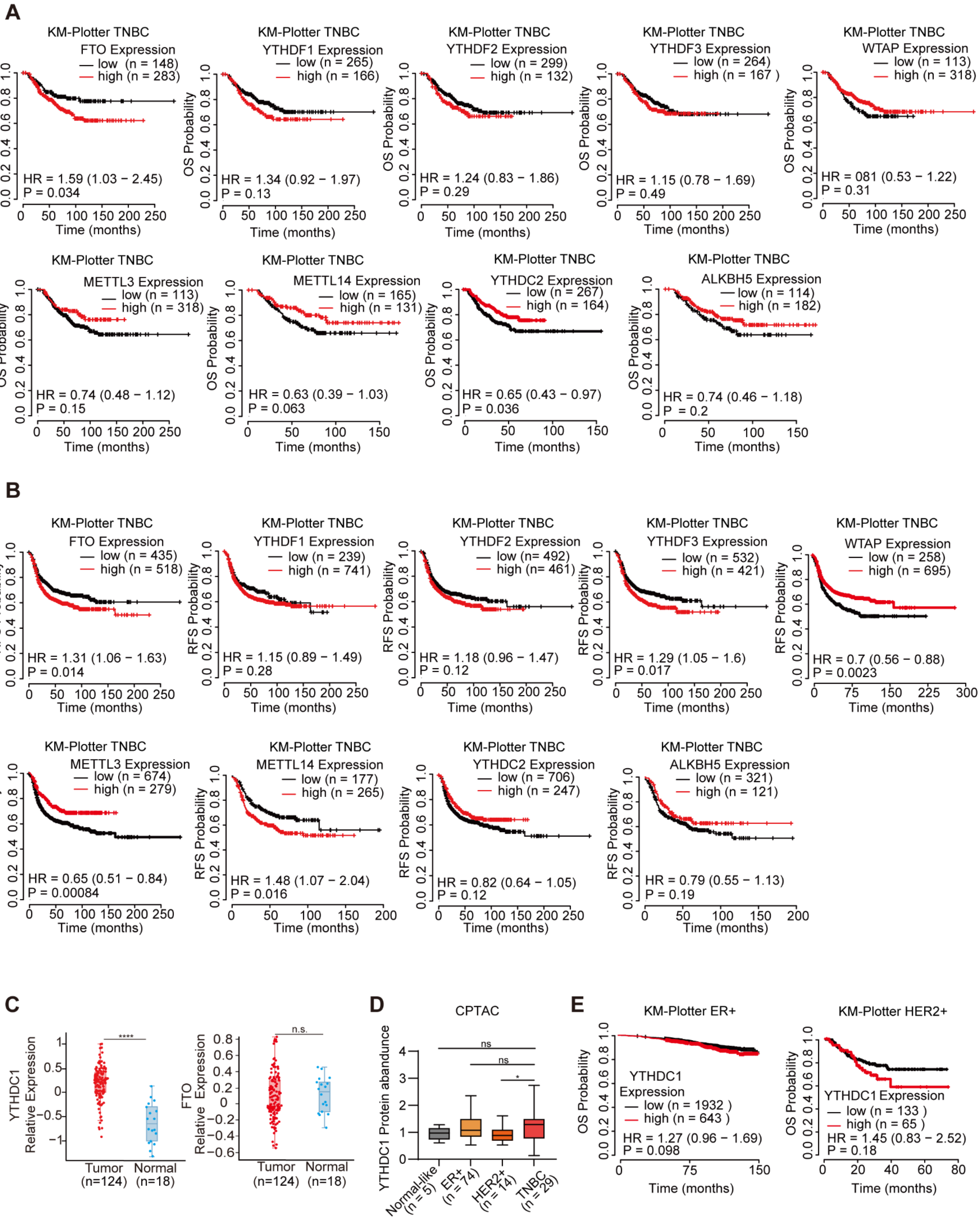

A
KM-Plotter TNBC
FTO Expression
low (n = 148)
high (n = 283)
HR = 1.59 (1.03 − 2.45)
P = 0.034
KM-Plotter TNBC
YTHDF1 Expression
low (n = 265)
high (n = 166)
HR = 1.34 (0.92 − 1.97)
P = 0.13
KM-Plotter TNBC
YTHDF2 Expression
low (n = 299)
high (n = 132)
HR = 1.24 (0.83 − 1.86)
P = 0.29
KM-Plotter TNBC
YTHDF3 Expression
low (n = 264)
high (n = 167)
HR = 1.15 (0.78 − 1.69)
P = 0.49
KM-Plotter TNBC
WTAP Expression
low (n = 113)
high (n = 318)
HR = 081 (0.53 − 1.22)
P = 0.31
KM-Plotter TNBC
METTL3 Expression
low (n = 113)
high (n = 318)
HR = 0.74 (0.48 − 1.12)
P = 0.15
KM-Plotter TNBC
METTL14 Expression
low (n = 165)
high (n = 131)
HR = 0.63 (0.39 − 1.03)
P = 0.063
KM-Plotter TNBC
YTHDC2 Expression
low (n = 267)
high (n = 164)
HR = 0.65 (0.43 − 0.97)
P = 0.036
KM-Plotter TNBC
ALKBH5 Expression
low (n = 114)
high (n = 182)
HR = 0.74 (0.46 − 1.18)
P = 0.2
OS Probability
Time (months)
B
KM-Plotter TNBC
FTO Expression
low (n = 435)
high (n = 518)
HR = 1.31 (1.06 − 1.63)
P = 0.014
KM-Plotter TNBC
YTHDF1 Expression
low (n = 239)
high (n = 741)
HR = 1.15 (0.89 − 1.49)
P = 0.28
KM-Plotter TNBC
YTHDF2 Expression
low (n= 492)
high (n= 461)
HR = 1.18 (0.96 − 1.47)
P = 0.12
KM-Plotter TNBC
YTHDF3 Expression
low (n = 532)
high (n = 421)
HR = 1.29 (1.05 − 1.6)
P = 0.017
KM-Plotter TNBC
WTAP Expression
low (n = 258)
high (n = 695)
HR = 0.7 (0.56 − 0.88)
P = 0.0023
KM-Plotter TNBC
METTL3 Expression
low (n = 674)
high (n = 279)
HR = 0.65 (0.51 − 0.84)
P = 0.00084
KM-Plotter TNBC
METTL14 Expression
low (n = 177)
high (n = 265)
HR = 1.48 (1.07 − 2.04)
P = 0.016
KM-Plotter TNBC
YTHDC2 Expression
low (n = 706)
high (n = 247)
HR = 0.82 (0.64 − 1.05)
P = 0.12
KM-Plotter TNBC
ALKBH5 Expression
low (n = 321)
high (n = 121)
HR = 0.79 (0.55 − 1.13)
P = 0.19
RFS Probability
Time (months)
C
YTHDC1 Relative Expression
****
Tumor (n=124)
Normal (n=18)
FTO Relative Expression
n.s.
Tumor (n=124)
Normal (n=18)
D
CPTAC
ns
ns
*
YTHDC1 Protein abandunce
Normal-like (n = 5)
ER+ (n = 74)
HER2+ (n = 14)
TNBC (n = 29)
E
KM-Plotter ER+
YTHDC1 Expression
low (n = 1932)
high (n = 643)
HR = 1.27 (0.96 − 1.69)
P = 0.098
KM-Plotter HER2+
YTHDC1 Expression
low (n = 133)
high (n = 65)
HR = 1.45 (0.83 − 2.52)
P = 0.18
OS Probability
Time (months)

Figure S1. The predictive value of m6A modulators for TNBC patient outcome.

A, Correlation of the expression of major m6A writers, erasers, and readers with overall survival (OS) from TNBC patients in the Kaplan-Meier plotter database. **B,** Correlation of the expression of major m6A writers, erasers, and readers with progression-free survival (PFS) from TNBC patients in the Kaplan-Meier plotter database. **C,** Quantitative mass spectrometry data from the Clinical Proteomic Tumor Analysis Consortium (CPTAC) demonstrate that YTHDC1 protein is significantly upregulated in breast cancer tissues compared to adjacent normal tissues, while FTO protein levels show no significant difference. **D,** CPTAC data of YTHDC1 in normal-like, ER+, HER2+, and TNBC subtypes of breast cancer. **E,** Correlation of YTHDC1 expression with OS in ER+ and HER2+ patients in the Kaplan-Meier plotter database. Data are presented as mean ± SD of samples (C, D). P values were assessed with Kaplan-Meier survival analysis (A-B, E) and two-tailed Student's t-test (C, D).

Figure S2

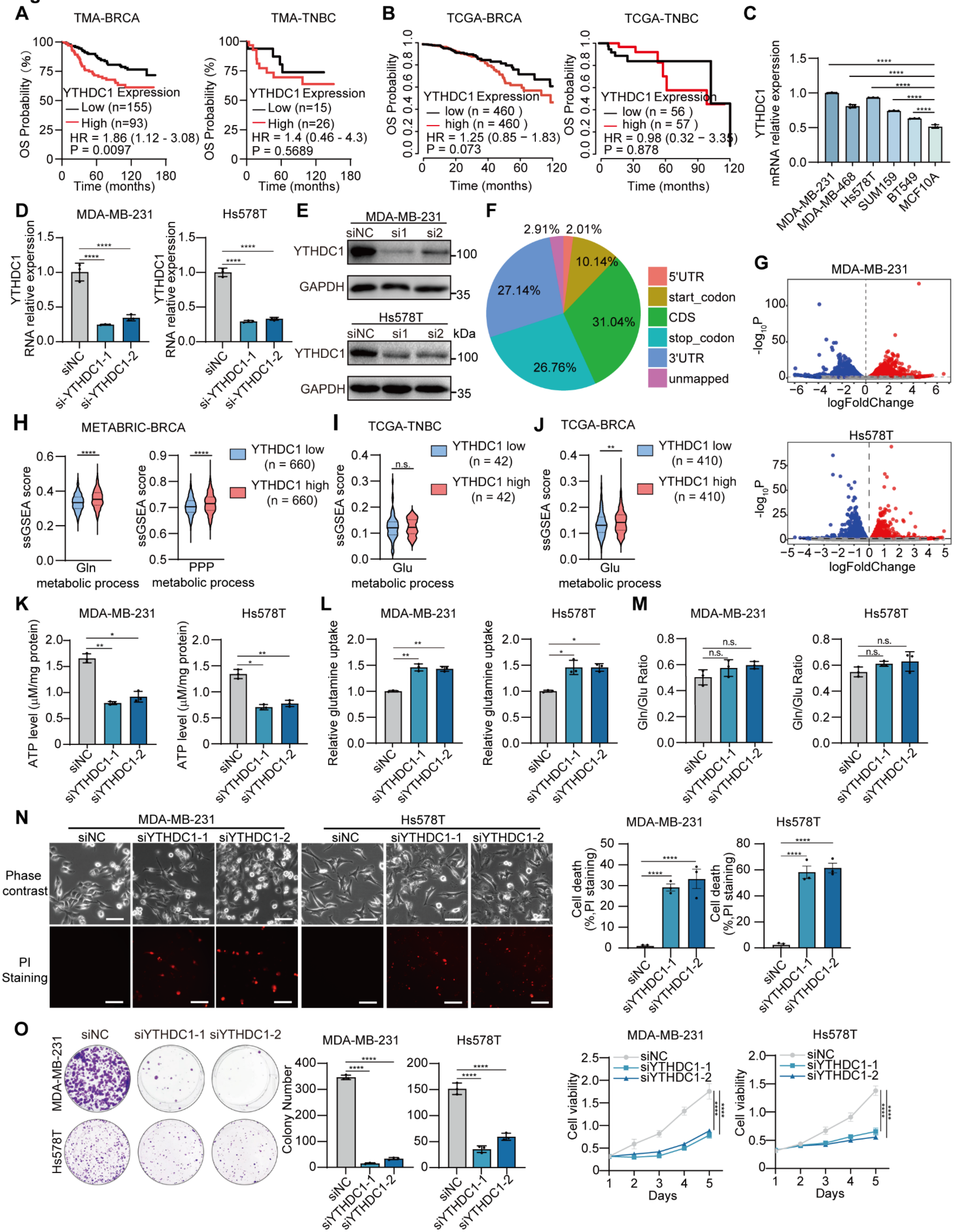

A
TMA-BRCA
TMA-TNBC
OS Probability (%)
YTHDC1 Expression
Low (n=155)
High (n=93)
HR = 1.86 (1.12 - 3.08)
P = 0.0097
Low (n=15)
High (n=26)
HR = 1.4 (0.46 - 4.3)
P = 0.5689
Time (months)
B
TCGA-BRCA
TCGA-TNBC
OS Probability
YTHDC1 Expression
low (n = 460)
high (n = 460)
HR = 1.25 (0.85 − 1.83)
P = 0.073
low (n = 56)
high (n = 57)
HR = 0.98 (0.32 − 3.35)
P = 0.878
Time (months)
C
YTHDC1 mRNA relative expression
MDA-MB-231
MDA-MB-468
Hs578T
SUM159
BT549
MCF10A
D
MDA-MB-231
Hs578T
YTHDC1 RNA relative experssion
siNC
si-YTHDC1-1
si-YTHDC1-2
E
MDA-MB-231
siNC
si1
si2
YTHDC1
GAPDH
Hs578T
kDa
100
35
F
2.91%
2.01%
10.14%
27.14%
31.04%
26.76%
5'UTR
start_codon
CDS
stop_codon
3'UTR
unmapped
G
MDA-MB-231
Hs578T
-log10P
logFoldChange
H
METABRIC-BRCA
ssGSEA score
YTHDC1 low (n = 660)
YTHDC1 high (n = 660)
Gln metabolic process
PPP metabolic process
I
TCGA-TNBC
n.s.
YTHDC1 low (n = 42)
YTHDC1 high (n = 42)
Glu metabolic process
J
TCGA-BRCA
YTHDC1 low (n = 410)
YTHDC1 high (n = 410)
Glu metabolic process
K
MDA-MB-231
Hs578T
ATP level (μM/mg protein)
siNC
siYTHDC1-1
siYTHDC1-2
L
Relative glutamine uptake
M
Gln/Glu Ratio
n.s.
N
MDA-MB-231
Hs578T
siNC
siYTHDC1-1
siYTHDC1-2
Phase contrast
PI Staining
Cell death (%,PI staining)
O
MDA-MB-231
Hs578T
Colony Number
Cell viability
Days

Figure S2. Multi-omics sequencing and functional analysis of YTHDC1.

A, Correlation of YTHDC1 expression with OS from all subtypes (left) and TNBC (right) from breast cancer tissue microarray (TMA). **B,** Correlation of YTHDC1 expression with OS from all subtypes (left) and TNBC (right) patients in the TCGA breast cancer (BRCA) database. **C,** YTHDC1 mRNA relative expression across different TNBC cells and non-tumorigenic MCF10A cells. **D, E,** RT-qPCR (D) and Western blot (E) confirm the efficient knockdown of YTHDC1 in MDA-MB-231 and Hs578T cells. **F**, Genomic distribution of YTHDC1 RIP-seq peaks, demonstrating a primary enrichment in coding sequences (CDS). **G**, Volcano plot showing the number of differentially expressed mRNAs identified by RNA-seq in two TNBC cell lines (MDA-MB-231 and Hs578T) following YTHDC1 KD. **H–J,** High YTHDC1 expression is associated with increased ssGSEA scores of Gln metabolism and PPP activity in METABRIC breast cancer cohort (METABRIC-BRCA) (H), and with increased ssGSEA scores of Glu metabolism in TNBC samples from the TCGA database (TCGA-TNBC) (I) and all breast cancer samples from the TCGA database (TCGA-BRCA) (J). **K, L**, **M**, Measurement of ATP level (K), glutamine (Gln) uptake (L) and the glutamate-to-glutamine (Glu/Gln) ratio (M) in control and YTHDC1 knockdown cells. **N**, PI staining demonstrates that YTHDC1 knockdown significantly increases the rate of cell death. Scale bars, 100 μm. **O**, Silencing YTHDC1 drastically reduces the growth of TNBC cells, as measured by colony formation (Left), and CCK-8 assays (Right). Data are presented as mean ± SD of experimental triplicates (A, D, F, G-I). P values were determined using two-tailed Student's t-test. (*$p < 0.05$, **$p < 0.01$, ***$p < 0.001$, **** $p < 0.0001$, n.s means not significant).

Figure S3

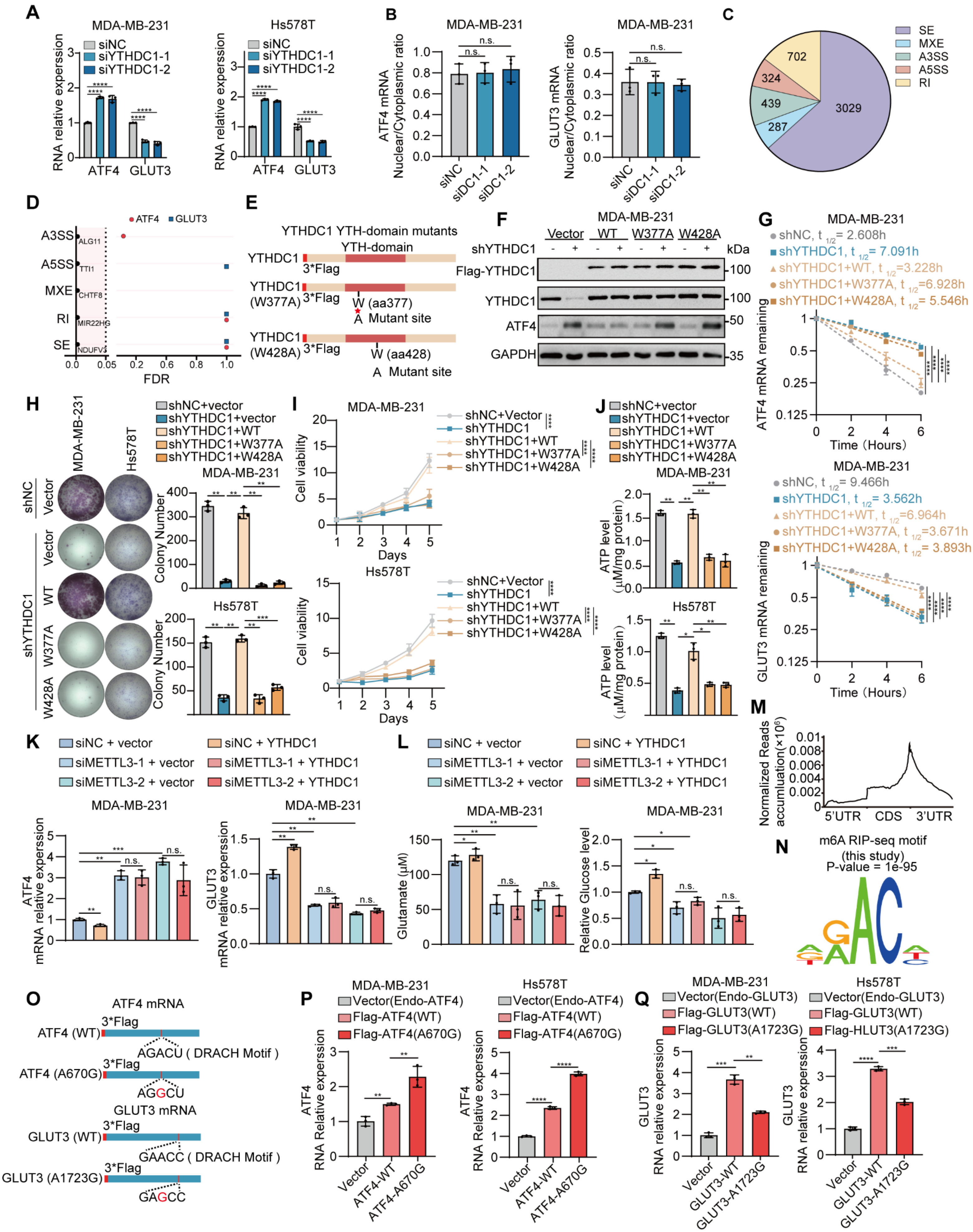

A
MDA-MB-231
siNC
siYTHDC1-1
siYTHDC1-2
RNA relative expression
ATF4
GLUT3
Hs578T
B
MDA-MB-231
ATF4 mRNA Nuclear/Cytoplasmic ratio
GLUT3 mRNA Nuclear/Cytoplasmic ratio
n.s.
siNC
siDC1-1
siDC1-2
C
SE
MXE
A3SS
A5SS
RI
702
324
439
287
3029
D
ATF4
GLUT3
A3SS
A5SS
MXE
RI
SE
ALG11
TTI1
CHTF8
MIR22HG
NDUFV3
FDR
E
YTHDC1 YTH-domain mutants
YTH-domain
YTHDC1
3*Flag
YTHDC1 (W377A)
W (aa377)
A Mutant site
YTHDC1 (W428A)
W (aa428)
A Mutant site
F
MDA-MB-231
Vector
WT
W377A
W428A
shYTHDC1
kDa
Flag-YTHDC1
YTHDC1
ATF4
GAPDH
100
50
35
G
MDA-MB-231
shNC, t 1/2= 2.608h
shYTHDC1, t 1/2= 7.091h
shYTHDC1+WT, t 1/2=3.228h
shYTHDC1+W377A, t 1/2=6.928h
shYTHDC1+W428A, t 1/2= 5.546h
ATF4 mRNA remaining
Time (Hours)
shNC, t 1/2= 9.466h
shYTHDC1, t 1/2= 3.562h
shYTHDC1+WT, t 1/2=6.964h
shYTHDC1+W377A, t 1/2=3.671h
shYTHDC1+W428A, t 1/2= 3.893h
GLUT3 mRNA remaining
H
MDA-MB-231
Hs578T
shNC
Vector
shYTHDC1
WT
W377A
W428A
shNC+vector
shYTHDC1+vector
shYTHDC1+WT
shYTHDC1+W377A
shYTHDC1+W428A
Colony Number
I
MDA-MB-231
Hs578T
shNC+Vector
shYTHDC1
shYTHDC1+WT
shYTHDC1+W377A
shYTHDC1+W428A
Cell viability
Days
J
ATP level (μM/mg protein)
K
siNC + vector
siMETTL3-1 + vector
siMETTL3-2 + vector
siNC + YTHDC1
siMETTL3-1 + YTHDC1
siMETTL3-2 + YTHDC1
ATF4 mRNA relative expression
GLUT3 mRNA relative expression
L
Glutamate (μM)
Relative Glucose level
M
Normalized Reads accumulation(×10^6)
5'UTR
CDS
3'UTR
N
m6A RIP-seq motif (this study)
P-value = 1e-95
O
ATF4 mRNA
ATF4 (WT)
AGACU (DRACH Motif)
ATF4 (A670G)
AGGCU
GLUT3 mRNA
GLUT3 (WT)
GAACC (DRACH Motif)
GLUT3 (A1723G)
GAGCC
P
MDA-MB-231
Hs578T
Vector(Endo-ATF4)
Flag-ATF4(WT)
Flag-ATF4(A670G)
ATF4 RNA Relative expression
Vector
ATF4-WT
ATF4-A670G
Q
Vector(Endo-GLUT3)
Flag-GLUT3(WT)
Flag-GLUT3(A1723G)
Flag-HLUT3(A1723G)
GLUT3 RNA relative expression
GLUT3-WT
GLUT3-A1723G

Figure S3. The m6A-binding capacity of YTHDC1 is required for its oncogenic functions.

A, RT-qPCR analysis of ATF4 and GLUT3 mRNA expression upon YTHDC1 knockdown (KD), showing opposing effects on their expression. **B,** YTHDC1 knockdown in TNBC cells didn't alter the nuclear/cytoplasmic distribution of ATF4 and GLUT3 mRNAs. **C,** Splicing events including skipped exons (SE), alternative 5'/3' splice sites (A3SS/A5SS), mutually exclusive exons (MXE), and retained introns (RI) were detected upon YTHDC1 knockdown, compared to siNC. **D,** No significant splicing event were detected in either ATF4 or GLUT3 transcripts upon YTHDC1 knockdown. **E,** Schematic of the wild-type (WT) YTHDC1 and its m6A-binding-deficient mutants (W377A, W428A). **F, G,** Ectopic expression of WT, but not mutant YTHDC1 reverses the mRNA stability of ATF4 and GLUT3 in YTHDC1-KD cells (G). **H-J,** Rescue experiments showing that WT, but not mutant YTHDC1 reverses colony formation (H), cell viability (I) and ATP levels (J) in YTHDC1-KD TNBC cells. **K,** Overexpression of YTHDC1 fails to restore RNA expression of ATF4 (left) and GLUT3 (right) in METTL3-knockdown MDA-MB-231 cells. **L,** Overexpression of YTHDC1 fails to restore intracellular glutamate levels (left) and glucose levels (right) in METTL3-knockdown MDA-MB-231 cells. **M,** Genomic distribution of YTHDC1 m6A-seq peaks, demonstrating a primary enrichment in CDS and 3'UTR. **N**, Schematic diagram showing the m6A modification sites in mRNA identified by m6A-seq data. **O**, Schematic representation of wild-type (WT) ATF4, ATF4 A670G mutant, WT GLUT3, and GLUT3 A1723G mutant. **P,** RT-qPCR using primers with or without a Flag-tag sequence to detect ATF4 mRNA expression in cells expressing vector control (endogenous ATF4), Flag-WT or A670G mutant ATF4. **Q,** RT-qPCR using primers with or without a Flag-tag sequence to detect GLUT3 mRNA expression in cells expressing vector control (endogenous GLUT3), Flag-WT or A1723G mutant GLUT3. Data are presented as mean ± SD of experimental triplicates (A, C, E, F). p values were determined using two-tailed Student's t-test. (*$p < 0.05$, **$p < 0.01$, ***$p < 0.001$, **** $p < 0.0001$).

Figure S4

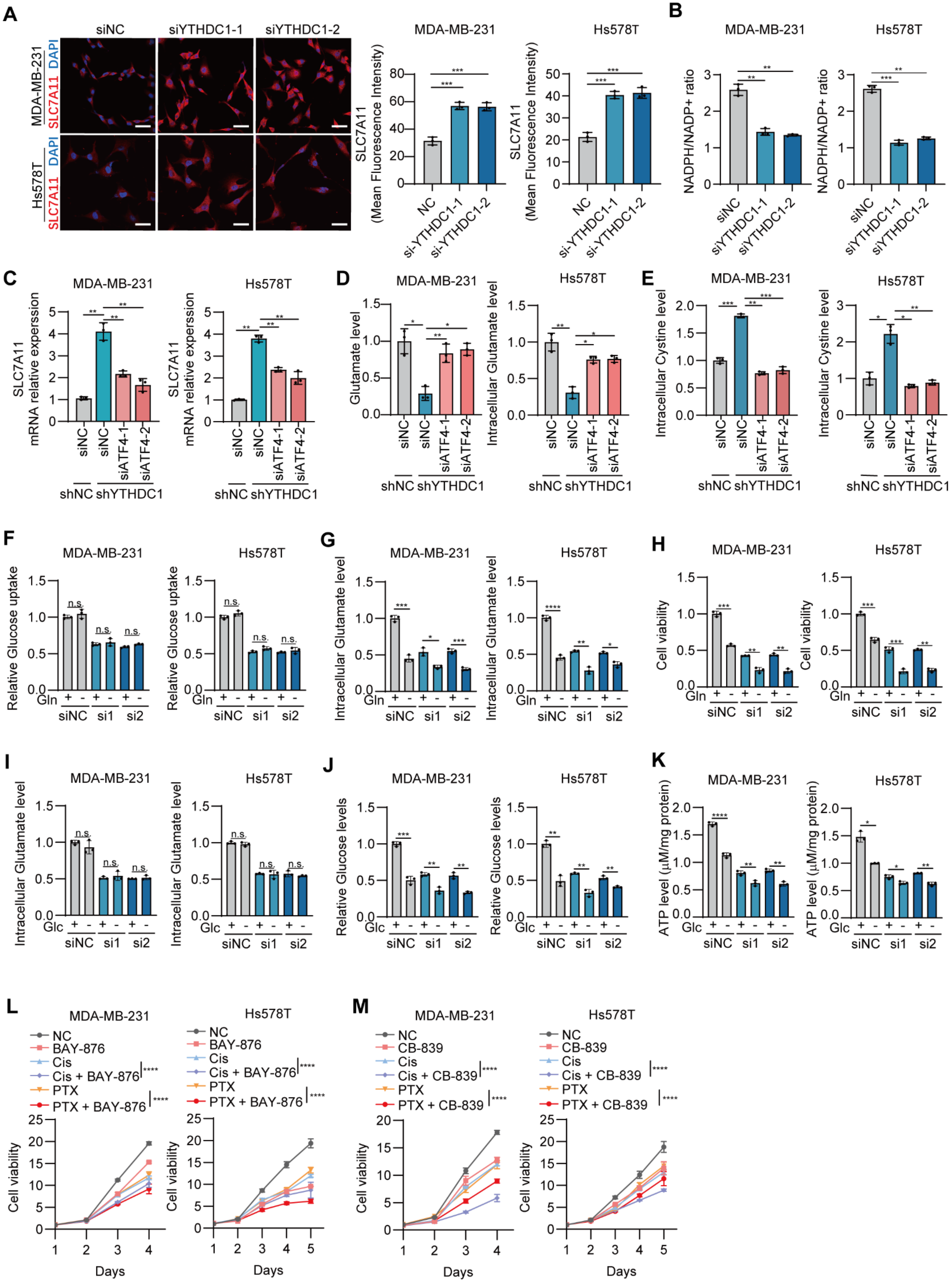

A
siNC
siYTHDC1-1
siYTHDC1-2
MDA-MB-231 SLC7A11 DAPI
Hs578T SLC7A11 DAPI
MDA-MB-231
Hs578T
SLC7A11 (Mean Fluorescence Intensity)
NC
si-YTHDC1-1
si-YTHDC1-2
B
NADPH/NADP+ ratio
C
SLC7A11 mRNA relative expression
siATF4-1
siATF4-2
shNC
shYTHDC1
D
Glutamate level
Intracellular Glutamate level
E
Intracellular Cystine level
F
Relative Glucose uptake
Gln
si1
si2
n.s.
G
H
Cell viability
I
Glc
J
Relative Glucose levels
K
ATP level (μM/mg protein)
L
NC
BAY-876
Cis
Cis + BAY-876
PTX
PTX + BAY-876
Days
M
CB-839
Cis + CB-839
PTX + CB-839

Figure S4. Knockdown of ATF4 rescues the disulfidptosis phenotype induced by YTHDC1 deficiency.

A, Immunofluorescence staining confirms the upregulation of SLC7A11 protein upon YTHDC1 knockdown in TNBC cells. Scale bars, 50 µm. **B**, YTHDC1-knockdown TNBC cells exhibit decreased NADPH/NADP$^+$ ratio. **C**, Knockdown of ATF4 rescue the expression of SLC7A11 in YTHDC1-knockdown TNBC cells. **D, E**, Intracellular glutamate (D) and Cystine levels (E) in YTHDC1-KD TNBC cells are rescued with siATF4. Scale bars, 20 µm. **F,** Glucose uptake measured by WST-8 assay in MDA-MB-231 and Hs578T cells under glutamine deprivation following YTHDC1 knockdown (siYTHDC1) versus siNC. YTHDC1 deficiency does not further alter glucose uptake under glutamine-depleted conditions. **G, H,** Intracellular glutamate levels (G) and cell viability (H) in MDA-MB-231 and Hs578T cells under glutamine deprivation following YTHDC1 knockdown (siYTHDC1) versus siNC. YTHDC1 knockdown exacerbates intracellular glutamate depletion and cell viability loss. **I,** Intracellular glutamate levels in MDA-MB-231 and Hs578T cells cultured in glucose-deficient medium following YTHDC1 knockdown (siYTHDC1) compared to siNC. YTHDC1 knockdown does not further affect glutamate levels under glucose deprivation. **J, K,** Intracellular glucose levels (J) and ATP levels (K) in MDA-MB-231 and Hs578T cells under glutamine deprivation following YTHDC1 knockdown (siYTHDC1) versus siNC. YTHDC1 knockdown exacerbates intracellular glucose and ATP depletion. **L,** CCK-8 assay of MDA-MB-231 and Hs578T cells treated with GLUT1/3 inhibitor BAY-876 (25 nM, 24h), cisplatin (Cis) (20 µM, 24h), paclitaxel (PTX) (15 pM, 24h), or their combinations. **M,** CCK-8 assay of MDA-MB-231 and Hs578T cells treated with glutaminase inhibitor CB-839 (500 nM, 24h), cisplatin (Cis) (20 µM, 24h), paclitaxel (PTX) (15 pM, 24h), or their combinations. Data are presented as mean ± SD of experimental triplicates. p values were determined using two-tailed Student's t-test. (*$p < 0.05$, **$p < 0.01$, ***$p < 0.001$, **** $p < 0.0001$).

Figure S5

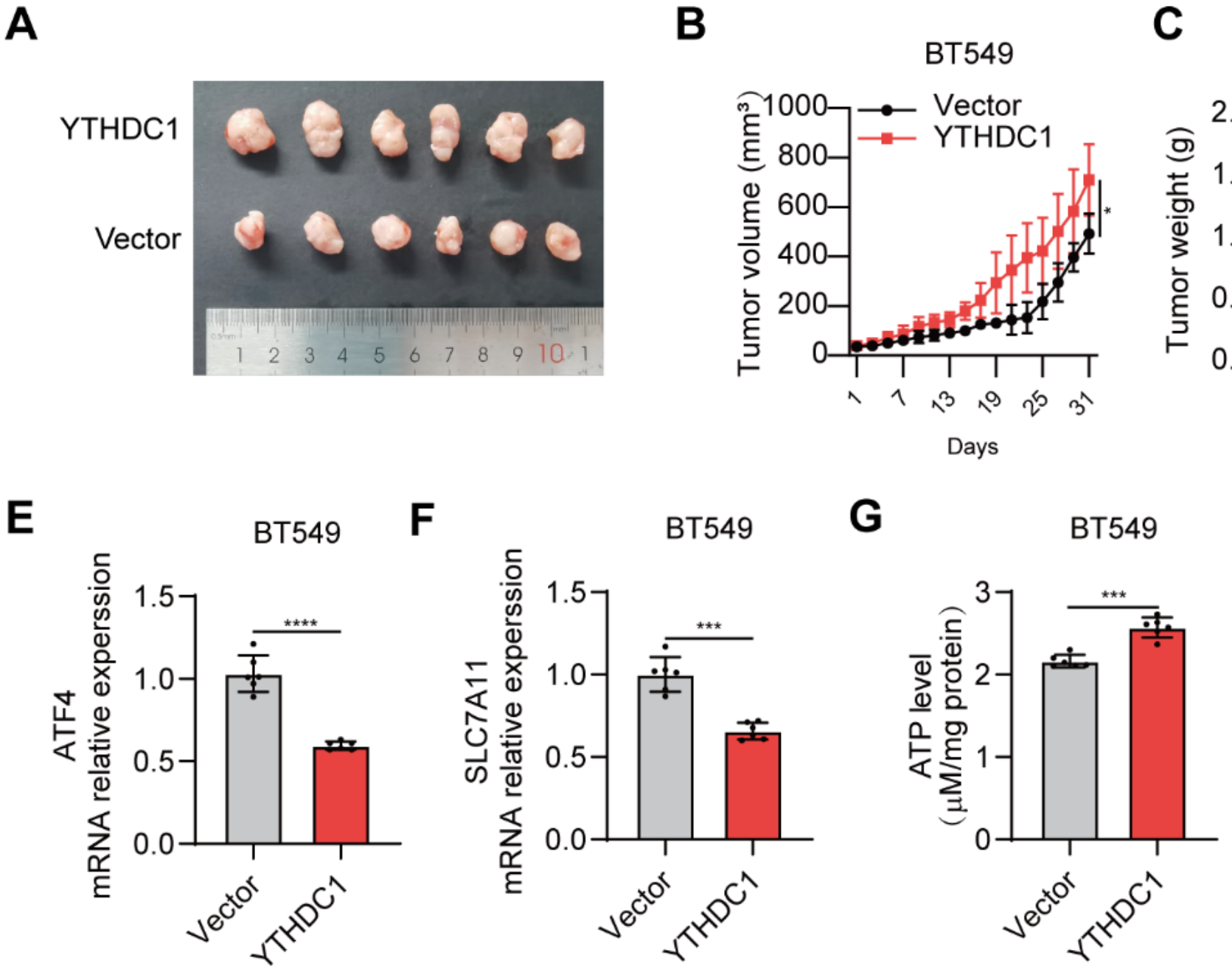

A
YTHDC1
Vector
B
BT549
Tumor volume (mm³)
Vector
YTHDC1
Days
C
BT549
Tumor weight (g)
D
BT549
YTHDC1 mRNA relative experssion
E
BT549
ATF4 mRNA relative experssion
F
BT549
SLC7A11 mRNA relative experssion
G
BT549
ATP level (μM/mg protein)
Vector
YTHDC1

Figure S5. YTHDC1 overexpression promotes tumor growth *in vivo*.

A, The images of BT549 xenograft tumors in nude mice with overexpression of YTHDC1 or control. **B, C,** Growth curves and tumor weights demonstrating that YTHDC1 overexpression promotes tumor growth. **D,** RT-qPCR analysis confirms the downregulation of ATF4 (E) and SLC7A11 mRNA (F), and elevated ATP (G) upon YTHDC1 overexperssion (D) in xenograft tumors. For A-G, n = 6 mice per group. Each dot represents one mouse (C–G). For C-G, mean ± SD are shown, and *p* values were determined using two-tailed Student's t-test. (*$p < 0.05$, **$p < 0.01$, ***$p < 0.001$, **** $p < 0.0001$).

Figure S6

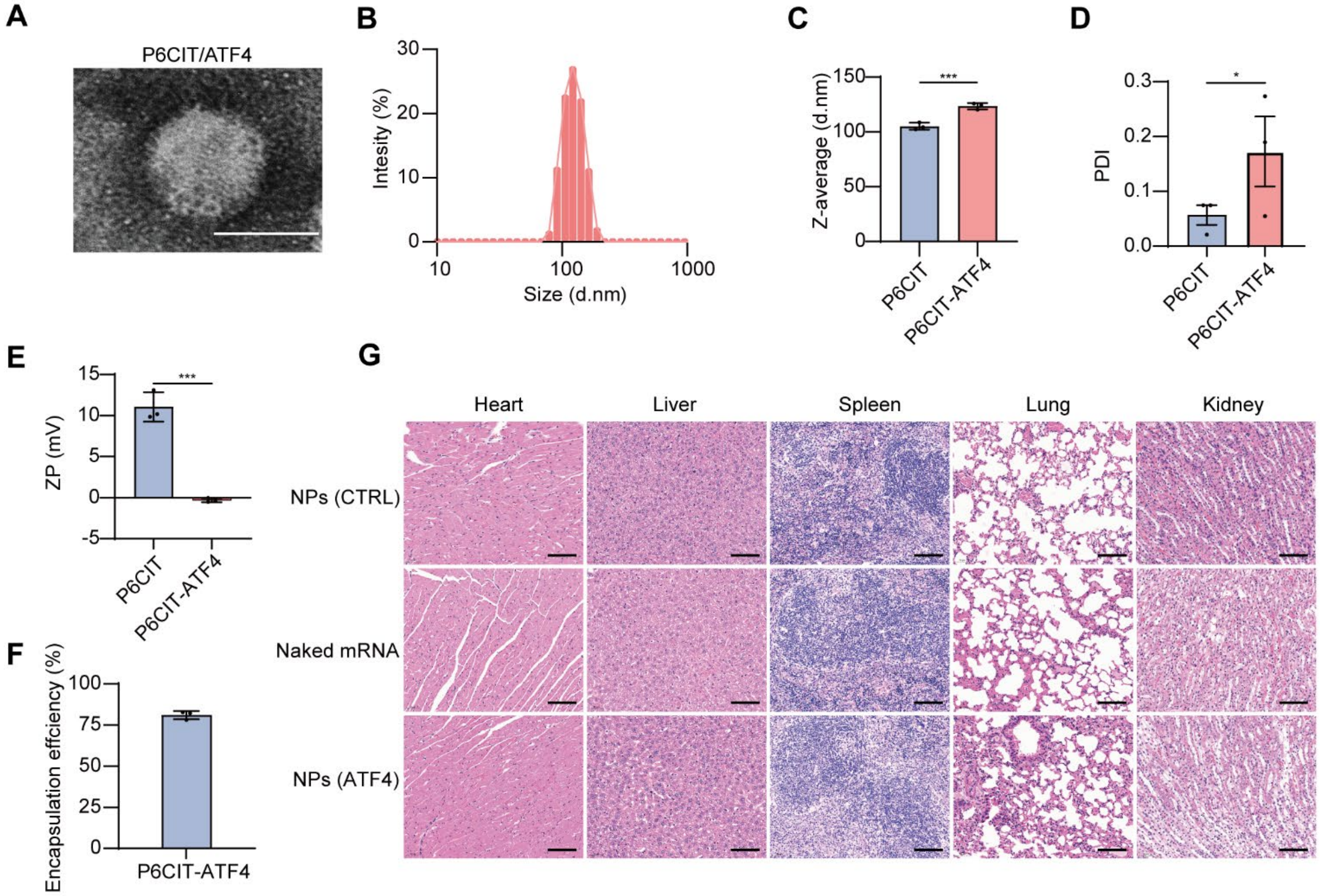

A
P6CIT/ATF4
B
Intesity (%)
Size (d.nm)
C
Z-average (d.nm)
***
P6CIT
P6CIT-ATF4
D
PDI
*
P6CIT
P6CIT-ATF4
E
ZP (mV)
***
P6CIT
P6CIT-ATF4
F
Encapsulation effciency (%)
P6CIT-ATF4
G
Heart
Liver
Spleen
Lung
Kidney
NPs (CTRL)
Naked mRNA
NPs (ATF4)

Figure S6. Characterization and functional validation of NPs (ATF4) in TNBC cells.

A, Morphological examination using transmission electron microscopy reveals NPs (ATF4) display homogeneous spherical morphology with narrow size distribution. Scale bars, 100 nm. **B, C,** Hydrodynamic size distribution analysis by dynamic light scattering shows NPs (ATF4) exhibits optimal nanoscale properties with a diameter of 123 ± 2.7 nm. **D,** Polydispersity index (PDI) determination indicates uniform size distribution of the nanoparticle formulation. **E,** Surface charge analysis through zeta potential measurement confirms neutral surface charge (−0.29 ± 0.24 mV) suitable for cellular delivery. **F,** mRNA encapsulation efficiency quantification demonstrates high loading capacity essential for effective gene delivery. **G**, H&E staining of major organs (heart, liver, spleen, lungs, and kidneys) reveals no significant histological abnormalities after NPs (ATF4) treatment. Scale bars, 100 μm. Data are presented as mean ± SD of experimental triplicates. *p* values were determined using two-tailed Student's *t*-test (*$p < 0.05$, **$p < 0.01$, ***$p < 0.001$, ****$p < 0.0001$).

Figure S7

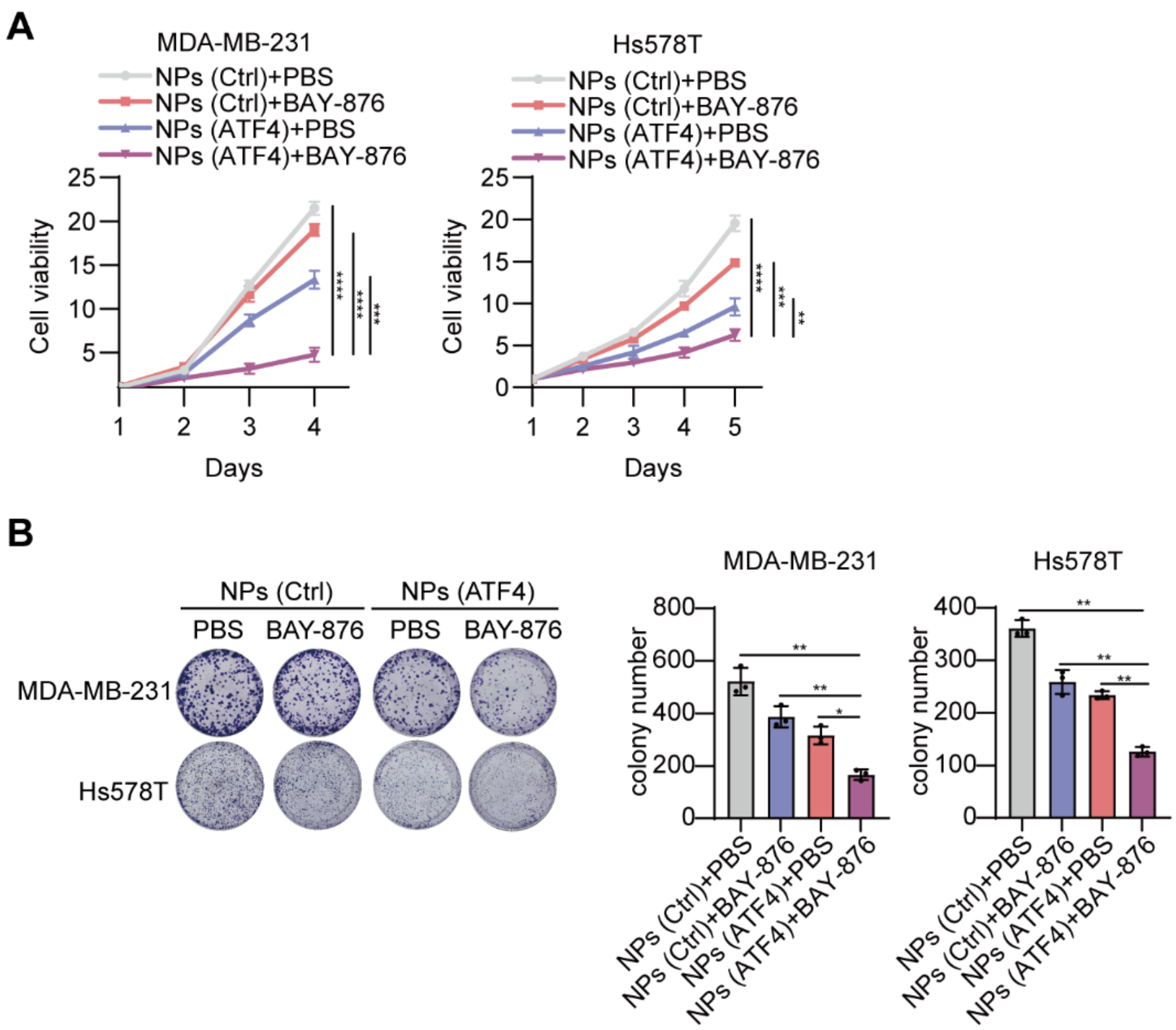
A
MDA-MB-231
NPs (Ctrl)+PBS
NPs (Ctrl)+BAY-876
NPs (ATF4)+PBS
NPs (ATF4)+BAY-876
Cell viability
Days
Hs578T
B
NPs (Ctrl)
NPs (ATF4)
PBS
BAY-876
MDA-MB-231
Hs578T
colony number

Figure S7. Combinatorial therapy of NPs (ATF4) and GLUTs inhibitor BAY-876 Inhibits cell proliferation and cell viability.

A, CCK-8 assay showing reduced cell viability with the combination therapy compared to either monotherapy. **B**, Colony formation assay reveals that combination therapy synergistically reduces colony numbers. Data are presented as mean ± SD of experimental triplicates. p values were determined using two-tailed Student's *t*-test (*$p < 0.05$, **$p < 0.01$, ***$p < 0.001$, ****$p < 0.0001$).

Table S1. Correlation between YTHDC1 IHC Staining and Clinical Characteristics.

	Number of samples	Staining index of YTHDC1		*P* value
		low (<5.0)	high (≥5)	
**Tumor size(cm)**				
Tumor Size（< 2cm）	39	30 (12.50%)	9 (3.75%)	0.0421
Tumor Size（≥2cm）	201	120 (50.00%)	81 (33.75%)	
**Stage**				
I-II	153	103 (42.92%)	50 (20.83%)	0.0408
III-IV	87	47 (19.58%)	40 (16.67%)	
**Grade**				
I-II	166	111 (46.25%)	55 (22.92%)	0.0363
III	74	39 (16.25%)	35 (14.58%)	
**Lymph Node Metastasis**				
negative	99	68 (28.33%)	31 (12.92%)	0.0971
positive	141	82 (34.17%)	59 (24.58%)	
**Ki67 (% of tumor cells)**				
≤20	181	120 (50.00%)	61 (25.42%)	0.0333
>20	59	30 (12.50%)	29 (12.08%)	

Table S2. ATF4 m6A modification site prediction by SRAMP.

#	Position	Seq-context	Structural-context	Score (binary)	Score (knn)	Score (spectrum)	Score (combined)	Decision
1	622	UGACC	IPPPP	0.72	0.346	0.826	0.744	m6A site (Very high confidence)
2	670	AGACU	PPPMM	0.844	0.527	0.822	0.819	m6A site (Very high confidence)
3	869	AGACU	PPHHH	0.804	0.583	0.822	0.8	m6A site (Very high confidence)

Table S3. GLUT3 m6A modification site prediction by SRAMP.

#	Position	Seq-context	Structural-context	Score (binary)	Score (knn)	Score (spectrum)	Score (combined)	Decision
1	1522	GGACU	PPIIP	0.775	0.589	0.687	0.73	m6A site (Very high confidence)
2	1723	GAACC	PPIPP	0.768	0.359	0.855	0.782	m6A site (Very high confidence)

Table S4. siRNA/shRNA sequences.

siRNAs and shRNAs	Target sequences
siYTHDC1-1	GCAAGGAGTGTTATCTTAA
siYTHDC1-2	GGCGTCGACCAGAAGATTA
siATF4-1	GAAGGAGTTCGACTTGGAT
siATF4-2	GTTGGATGACACTTGTGAT
siMETTL3-1	ACUUCUUCUCUAAUUCAGGGU
siMETTL3-2	GCAACCCGCAAGGCUUCAATT
shYTHDC1	GCAAGGAGTGTTATCTTAA

Table S5. Primers for RT-qPCR.

Gene name	Sequence
YTHDC1-Forward	TCAGGAGTTCGCCGAGATGTGT
YTHDC1-Reverse	AGGATGGTGTGGAGGTTGTTCC
ATF4-Forward	TTCTCCAGCGACAAGGCTAAGG
ATF4-Reverse	CTCCAACATCCAATCTGTCCCG
GLUT3-Forward	TGCCTTTGGCACTCTCAACCAG
GLUT3-Reverse	GCCATAGCTCTTCAGACCCAAG
SLC7A11-Forward	TCCTGCTTTGGCTCCATGAACG
SLC7A11-Reverse	AGAGGAGTGTGCTTGCGGACAT
GAPDH-Forward	GTCTCCTCTGACTTCAACAGCG
GAPDH-Reverse	ACCACCCTGTTGCTGTAGCCAA
Flag-ATF4-Forward	GACTACAAAGACCATGACGGTG
Flag-ATF4-Reverse	ATCAAGTCCCCCACCAACAC
Flag-GLUT3-Forward	TCGGAGGTGGAGGATCAGAC
Flag- GLUT3-Reverse	CCTTGTTGAGGGAGAGGTGG
